# HDAC1/3-dependent moderate liquid–liquid phase separation of YY1 promotes METTL3 expression and AML cell proliferation

**DOI:** 10.1038/s41419-022-05435-y

**Published:** 2022-11-24

**Authors:** Meng Li, Mingying Li, Yuan Xia, Guosheng Li, Xiuhua Su, Dongmei Wang, Jingjing Ye, Fei Lu, Tao Sun, Chunyan Ji

**Affiliations:** 1 Department of Hematology, Qilu Hospital of Shandong University, Cheeloo College of Medicine, Shandong University, Jinan, 250012 People’s Republic of China; 2 Shandong Key Laboratory of Immunohematology, Qilu Hospital of Shandong University, Cheeloo College of Medicine, Shandong University, Jinan, 250012 People’s Republic of China

**Keywords:** Acute myeloid leukaemia, Mechanisms of disease, Transcriptional regulatory elements, DNA-binding proteins

## Abstract

Methyltransferase-like protein 3 (METTL3) plays critical roles in acute myeloid leukemia (AML) progression, however, the mechanism of abnormal overexpression of METTL3 in AML remain elusive. In the current study, we uncovered that Yin Yang 1 (YY1) binds to the promoter region of METTL3 as a transcription factor and promotes its expression, which in turn enhances the proliferation of AML cells. Mechanistically, YY1 binds to HDAC1/3 and regulates METTL3 expression in a moderate liquid-liquid phase separation (LLPS) manner. After mutation of the HDAC-binding site of YY1 or HDAC inhibitor (HDACi) treatment, YY1 was separated from HDAC1/3, which resulted in an excessive LLPS state, thereby inhibiting the expression of METTL3 and the proliferation of AML cells. In conclusion, our study clarified the regulatory mechanism of the abnormal expression of METTL3 in AML, revealed the precise “Yin-Yang” regulatory mechanism of YY1 from the perspective of LLPS degree, and provided new ideas for the precise diagnosis and treatment of AML.

## Introduction

Acute myeloid leukemia (AML) is a heterogeneous hematological malignancy characterized by accumulation of immature cells and blocked differentiation, and is the most common acute leukemia in adults and generally has a poor prognosis [1–3]. Methyltransferase-like protein 3 (METTL3), also named MT-A70, was first identified in humans as an s-adenosylmethionine-binding protein [4, 5]. Recent studies have confirmed that METTL3 is abnormally overexpressed in AML and is closely related to the occurrence and development of AML [6]. Previous studies by our group found that METTL3 also plays an important role in the chemoresistance of AML [7]. A METTL3 inhibitor has shown promising therapeutic effects in AML animal models [8]. However, METTL3-mediated m^6^A modification plays an important role in the central and cardiac systems [9], and the prospect of systemic administration of its inhibitor is less clear. Therefore, the development of AML-specific targeted intervention mechanisms of METTL3 is very important for the application of METTL3 in the clinical treatment of AML. Studies have confirmed that METTL3 expression is regulated by different regulatory mechanisms in different tumors and organs. For example, in colorectal cancer, forkhead box D3 acts as a transcription factor of METTL3 and inhibits colorectal cancer metastasis by promoting METTL3 expression [10]. In pancreatic cancer, cigarette smoke condensate leads to hypomethylation of the METTL3 promoter, which promotes the recruitment of the transcription factor NFIC and induces METTL3 overexpression [11]. For gastric cancer, P300-mediated H3K27 acetylation activation in the METTL3 promoter induces METTL3 transcription [12]. In non-small-cell lung carcinoma, SUMOylation of METTL3 regulates its m^6^A methyltransferase activity [13]. The abnormal expression of METTL3 in different tumors suggests that METTL3 expression is finely regulated in different contexts. Elucidating the mechanism of abnormal overexpression of METTL3 in AML has become a new option for targeted intervention.

Yin Yang 1 (YY1) is a ubiquitous zinc-finger transcription factor that plays a dual role in transcriptional regulation [14, 15], and plays an important role in AML [16–18]. YY1 activates or represses gene transcription depending on the cellular context [19–21]. Firstly, as a traditional transcription factor, YY1 interacts with a wide range of cofactors. Such as the protein-protein interaction between YY1 and DNA-binding cofactors SpI synergistic activate transcription [22]. Meanwhile YY1 zinc-finger domain mediates the association of it with Smads and represses its transcription activities in a gene-specific manner [23]. Secondly, YY1 interacts with chromatin remodeling complexes to influence gene expression. For instance, YY1 recruits Polycomb complexes to specific DNA sites, initiates methylation of histone 3 lysine 27 (H3K27me3) and regulates transcription [24]. In contrast, YY1 recruits the methyltransferase PRMT1, which methylates histone 4 arginine 3, to promote transcription [25]. Hence, through a plethora of chromatin remodeling complexes interactions, YY1 influences chromatin modifications and ultimately gene expression. Thirdly, YY1 can activate expression by controlling chromatin loops, stabilizing enhancer-promoter interactions [19]. Analyzing the underlying mechanism by which YY1 realizes its complex regulatory role has long been a focus of researchers.

In the present study, we reported that YY1 was positively correlated with METTL3 in AML patients, and the moderate LLPS of YY1 enhanced the expression of METTL3. LLPS refers to the process by which key molecules aggregate with other proteins or RNAs into confined, liquid-like cavities when a threshold concentration is reached [26] and is involved in a variety of body pathological and physiological processes, such as immunity [27], transcription [28], autophagy [26], and the development of cancer [29] and neurodegenerative diseases [30]. LLPS can be regulated by a variety of factors, such as temperature [31], ubiquitination [32], acetylation [33], RNA [34], and DNA [35]. YY1 has been recently reported to be capable of LLPS to mediate the activation of YY1 function through its transcriptional activation domain [36]. In our study, we further found that moderate YY1 LLPS activated METTL3 expression. More importantly, we found that mutation of the HDAC-binding site in the YY1 inhibitory domain prevented HDAC1/3 binding, led to excessive LLPS of YY1 and inhibited its promotion of METTL3 expression. Our study re-understood the precise “Yin-Yang” regulatory mechanism of YY1 from the perspective of LLPS degree and provides new ideas for the precise diagnosis and treatment of AML.

## Materials and methods

### Patient samples

Bone marrow (BM) samples from 78 AML patients were obtained from the Qilu Hospital of Shandong University, China. Mononuclear cells were isolated and used for RT-qPCR analysis. Informed consent was obtained according to the Declaration of Helsinki. This study was approved by the Medical Ethics Committee of Qilu Hospital of Shandong University.

### Cell lines and cell culture

For cells, THP-1 and HEK293T cells were obtained from the Institute of Hematology & Blood Diseases Hospital, Chinese Academy of Medical Sciences & Peking Union Medical College, Tianjin, China; Kasumi-1 cells were obtained from DSMZ; U2OS cells were obtained from the Pathology Laboratory of Shandong University. THP-1 cells were cultured in RPMI-1640 medium with 10% fetal bovine serum (FBS)(Gibco) and 1% penicillin-streptomycin (Invitrogen); Kasumi-1 cells were cultured in IMDM with 20% FBS and 1% penicillin-streptomycin; U2OS cells were cultured in McCoy’s 5 A with 10% FBS and 1% penicillin-streptomycin, and HEK293T cells were cultured in DMEM with 10% FBS and 1% penicillin-streptomycin. All the cell lines were verified by short tandem repeat (STR) analysis within the past 6 months. Cells were routinely measured for Mycoplasma contamination.

### Chemical inhibitors

The histone deacetylase inhibitor chidamide (CHI; Selleck) was dissolved in DMSO (4 µM in culture).

### Cell transfection

THP-1, Kasumi-1 and HEK293T cells were transfected with the aid of Lipofectamine 2000 (Invitrogen). Cells were collected 24 h or 48 h after transfection and used for various purposes.

### Quantitative real-time PCR analysis

Total RNA was extracted using TRIzol (Invitrogen). The RNA samples were reverse-transcribed into cDNA with PrimeScript™ RT Master Mix (Takara). RT-qPCR was performed with TB Green®Premix Ex Taq™ II (Takara). The results were analyzed using the 2^-ΔΔCT^ method, and GAPDH was used as an internal control. The primers are listed in Supplementary Data 1.

### Western blot

Cells were lysed in M-PERTM Mammalian Protein Extraction Reagent (Thermo Fisher). Proteins were separated by 10% SDS-PAGE and transferred to nitrocellulose membranes. The membranes were blocked with 5% nonfat milk (Sangon Biotech) and then incubated successively with primary and secondary antibodies. Protein bands were incubated with HRP substrate luminol reagent (Millipore) and visualized using ChampChemi (SageCreation).

### Cell proliferation assay

For EdU assays, cells were seeded in 6-well plates at approximately 300,000 cells per well with or without 4 µM CHI for 24 h. The treated cells were analyzed with the iClickTM EdU Andy FluorTM 647 Flow Cytometry Assay Kit (GeneCopoeia) and tested by flow cytometry. For CCK-8 assays, cells were seeded in 96-well plates and cultured in an incubator. At the same time point in the following 3 days, 10 µL CCK-8 (BestBio) was added to each well, the cells were incubated for 4 h and the absorbance at 450 nm was measured by a microplate reader (Thermo Scientific). For the colony formation assay, cells were seeded in methylcellulose medium (MethoCult, StemCell Technologies). Colonies were counted after 2–3 weeks.

### Luciferase assay

HEK293T cells were plated in 24-well plates at 100,000 cells per well. HEK293T cells were cotransfected with 500 ng of WT or mutant firefly luciferase reporter vector (Supplemental Data 1), 500 ng of YY1 expression vector and 50 ng of pRL-TK Renilla luciferase reporter vector. Cells were incubated for 48 h and the luciferase activities were assessed using the Dual-Luciferase Reporter Assay System (Promega) following the manufacturer’s protocol.

### Chromatin immunoprecipitation

For the Chromatin immunoprecipitation (ChIP) assay, the SimpleChIP^®^ Enzymatic Chromatin IP Kit (Cell Signaling Technology) was used following the manufacturer’s protocol. Chromatin fragments derived from THP-1 cells were immunoprecipitated with 5 µg YY1 antibody (Abcam). The primers for the ChIP-PCR assay are listed in Supplementary Data 1.

### Coimmunoprecipitation

The YY1-EGFP expression vectors and HDAC1-HA or HDAC3-HA expression vectors were cotransfected into HEK293T cells. Cells were lysed with IP buffer (10 mM Tris pH8.0, 150 mM NaCl, 1% NP-40, 1 mM EDTA pH8.0, 10% glycerol). Total protein was incubated with anti-EGFP mAb-agarose (MBL) or anti-HA beads (Sigma). Captured agarose bead-Ab-Ag complexes were washed with IP buffer and detected by western blotting using anti-EGFP antibody (Invitrogen) and anti-HA antibody (Abways).

### Immunofluorescence

Cells were fixed with 4% paraformaldehyde for 15 min. Then the cells were permeabilized with 0.3% Triton X-100 for 20 min and blocked with 4% bovine serum albumin for 1 hour. Cells were incubated successively with primary antibody overnight at 4 °C and Alexa-Fluor-488- or 594-conjugated secondary antibody (Thermo Fisher Scientific) for 1 h at room temperature. Cells were examined with a ZESIS LSM980 confocal microscope.

### Colocalization assay

48 h after THP-1 or Kasumi-1 cells were transfected with HDAC1-HA/HDAC3-HA and treated with or without CHI for 1 h, the cells were collected for immunofluorescence experiments. Cells were incubated overnight in rabbit-derived anti-YY1 antibody (CST) and mouse-derived anti-HA antibody (PTG). This was followed by incubation with the fluorescent antibodies donkey anti-mouse IgG (H + L) Alexa 594 (Thermo Fisher) and donkey anti-rabbit IgG (H + L) Alexa 488 (Thermo Fisher) for 1 h. Cells were examined with a ZESIS LSM980 confocal microscope.

### Protein expression and purification

A bacterial expression plasmid for EGFP-YY1 was constructed by using the pET28a vector. The plasmid was transformed into *E. coli* BL21 cells and induced with isopropyl-β-d-thiogalactoside. The recombinant proteins were purified with Ni-NTA agarose beads (QIAGEN) washed with washing buffer (10 mM Tris-HCl pH 8.0, 300 mM NaCl, 100/250/500 mM imidazole), and then concentrated with centrifugal filter units (Millipore) according to the manufacturer’s protocols.

### Droplet formation and droplet fusion assay

One microliter of concentrated protein was added to 2 µl 4 M NaCl and 8 µl 10% PEG 8000. Droplets were formed in a glass-bottomed dish and images were taken using an LSM980 confocal microscope (Zesis). Droplet fusion in vitro was recorded with the an LSM980 confocal microscope with a 63× oil immersion objective.

### Fluorescence recovery after photobleaching

The droplets were bleached with a 488-nm laser until the region of interest had almost no fluorescence. Then the recovery of fluorescence after photobleaching was recorded.

### Statistical analyses

Data are presented as the mean ± SD. Unpaired Student’s *t* test was used to analyze the differences between two groups. One-way ANOVA or two-way ANOVA was used to compare differences between more than 3 groups. *P* < 0.05 was considered to indicate statistical significance.

### Additional materials and methods

Antibodies, primers, and interference sequences are provided in the Supplemental Data 1.

## Results

### YY1 enhances METTL3 expression as a transcription factor

**Fig. 1:**
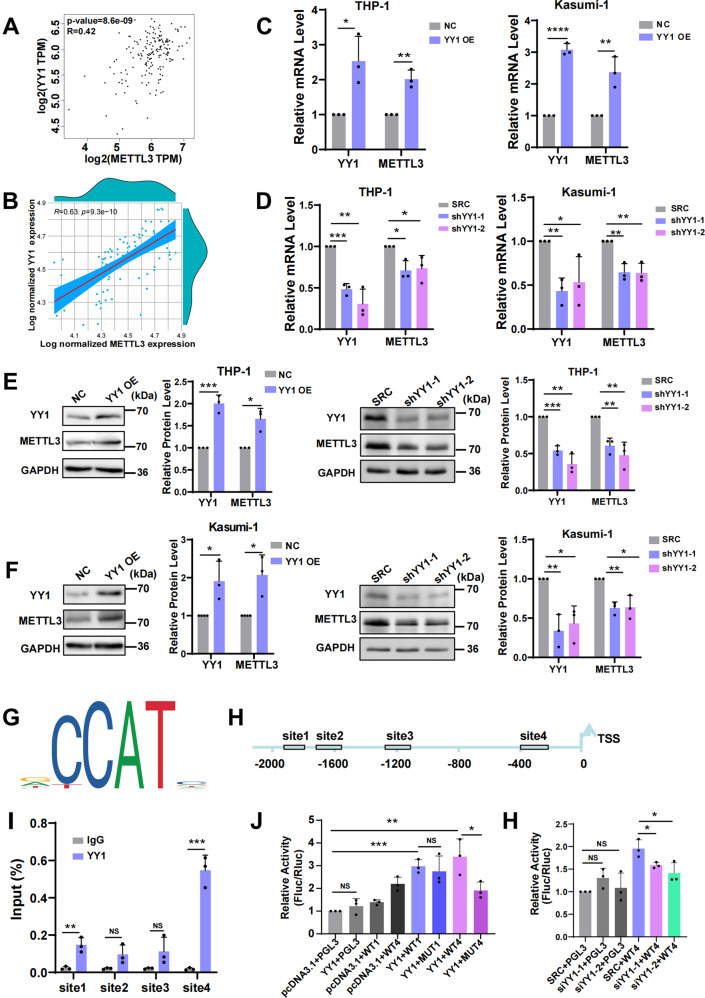
YY1 enhances METTL3 expression as a transcription factor. **A** Pearson correlation of YY1 with METTL3 in expression in AML samples from TCGA. **B** Pearson correlation of YY1 with METTL3 in expression in AML samples from Qilu Hospital of Shandong University as detected by qPCR. *n* = 78. **C** qPCR analysis of the expression of YY1 and METTL3 in THP-1 or Kasumi-1 cells transfected with LV5-homo-YY1 or LV5-NC. *P < 0.05; **P < 0.01; ****P < 0.0001, *t*-test. **D** qPCR analysis of the expression of YY1 and METTL3 in THP-1 or Kasumi-1 cells transfected with LV3-shYY1-1 or LV3-shYY1-2 or LV3-SRC. *P < 0.05; **P < 0.01; ***P < 0.001, *t*-test. **E**, **F** Western blotting analysis of the expression of YY1 and METTL3 in THP-1 cells (**E**) or Kasumi-1 cells (**F**) that overexpress or interfere YY1. *P < 0.05; **P < 0.01; ***P < 0.001, *t*-test. **G** The conservative binding motif of YY1 to the METTL3 promoter predicted by the JASPAR website. **H** Binding sites of YY1 to the METTL3 promoter region. **I** CHIP-qPCR experiments in THP-1 cells. **P < 0.01; ***P < 0.001; NS = nonsignificant, *t*-test. **J** Luciferase activity in HEK293T cells co-transfected with the YY1 overexpression plasmids and the luciferase reporter vectors assessed by luciferase reporter assay. *P < 0.05; **P < 0.01; ***P < 0.001; NS = nonsignificant, *t*-test. **H** Luciferase activity in HEK293T cells co-transfected with the YY1 siRNA and the luciferase reporter vectors assessed by luciferase reporter assay. *P < 0.05; NS = nonsignificant, *t*-test.

To further clarify the upstream regulatory mechanism of METTL3, we used the ChIPBase v2.0 website (http://rna.sysu.edu.cn/chipbase/) to predict the transcription factors of METTL3 and found that the transcription factor YY1 was the top transcription factor. Then, we noticed that YY1 was obviously enriched in the promoter region of the METTL3 gene through the UCSC website (www.genome.ucsc.edu). The Gene Expression Profiling Interactive Analysis (GEPIA) website (gepia.cancer-pku.cn) showed that YY1 is positively correlated with METTL3 in AML samples from The Cancer Genome Atlas (TCGA) database (Fig. 1A). We therefore hypothesized that YY1 is a transcription factor for METTL3. To test this hypothesis, we detected the RNA levels of YY1 and METTL3 in bone marrow mononuclear cells of AML patients, and the results showed that they were significantly positively correlated (Fig. 1B). Next, we transfected the human AML cell lines THP-1 and Kasumi-1 with lentivirus to overexpress or silence YY1 and used RT-qPCR and Western blotting to detect the transfection efficiency and the impact on METTL3 expression. We found that the changes in the expression of METTL3 were consistent with the changes in the expression of YY1 (Fig. 1C–F). To eliminate off-target effects, YY1 shRNA resistant plasmid and YY1 shRNA were co-transfected into the cells, and the changes of the expression of YY1 and METTL3 were verified (Fig. S1A, B). To confirm this hypothesis, we predicted the YY1 binding site motif approximately 2000 bp upstream of METTL3 ORF via the JASPAR website (https://jaspar.genereg.net) at first. YY1 was predicted to bind to the promoter sequence upstream of METTL3, and four pairs of primers were designed for four main regions with a dense distribution of this motif (Fig. 1G, H). Furthermore, ChIP-qPCR experiments showed that the enrichment level of the METTL3 promoter fragment pulled down using an anti-YY1 antibody was much higher than that using an anti-IgG antibody, especially at site 1 and site 4 (Fig. 1I). Then, as the previous reports [37, 38], we constructed four luciferase reporter vectors containing site 1 or site 4 or their mutants to further verify the binding site (Supplemental Data 1). We co-transfected HEK293T cells with the YY1 plasmid with the different luciferase reporter vectors found that luciferase activity was significantly decreased in HEK293T cells with site 4 mutation compared to wild-type cells (Fig. 1J). In addition, we interfered with endogenous YY1 for dual luciferase report assay and found that YY1 KD could significantly decreased the luciferase activity of cells (Fig. 1H). All these data suggested that YY1 positively regulates METTL3 expression in AML cells.

### YY1 enhances AML cell proliferation in a METTL3-dependent manner

**Fig. 2:**
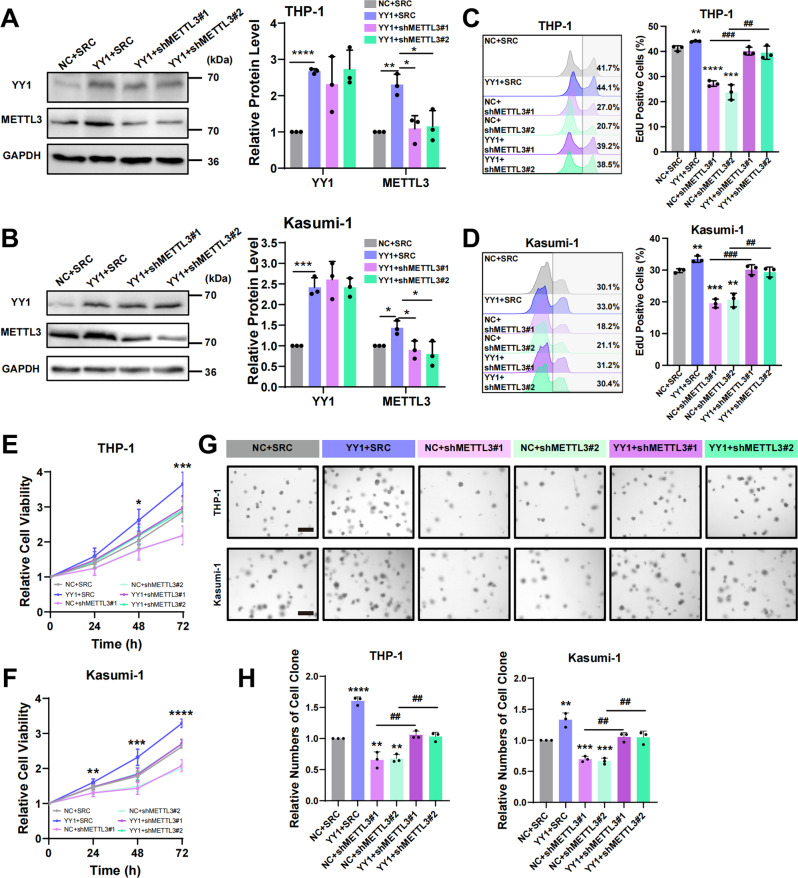
YY1 enhances AML cell proliferation in a METTL3-dependent manner. **A**, **B** Western blotting analysis of YY1 and METTL3 expression at protein level in YY1 OE or NC THP-1 (**A**) and Kasumi-1 (**B**) cells transfected with METTL3 interfering virus or scramble virus. *P < 0.05; **P < 0.01; ***P < 0.001; ****P < 0.0001, *t*-test. **C**, **D** The proliferative activity of THP-1 (**C**) and Kasumi-1 (**D**) cells measured by flow cytometry EdU assay. *Compare to the NC + SRC group. **P < 0.01; ***P < 0.001; ***P < 0.0001; ^##^P < 0.01; ^###^P < 0.001, *t*-test. **E**, **F** The proliferative activity of THP-1 (**E**) and Kasumi-1 (**F**) cells measured by CCK-8 assay. *P < 0.05; **P < 0.01; ***P < 0.001; ****P < 0.0001, one-way ANOVA. **G** The number of cell clone formed assessed by colony formation assay. Scale bars, 500 μm. **H** Statistical chart of colony formation experiment. *Compare to the NC + SRC group. **P < 0.01; ***P < 0.001; ***P < 0.0001; ^##^*P* < 0.01, *t*-test.

It had been reported [17] and been proved by us that YY1 enhanced the proliferation of AML cells (Fig. S1C-H). We constructed METTL3-interfering lentivirus and the extent of METTL3 knockdown was assessed by western blotting (Fig. S3A, B). To determine whether YY1 enhances AML cell proliferation by increasing METTL3 expression, we further transfected YY1-overexpressing THP-1 and Kasumi-1 cells with METTL3-interfering lentivirus and performed western blotting to detect YY1 and METTL3 expression in THP-1 and Kasumi-1 cells (Fig. 2A, B). By flow cytometry EdU assays, we found that overexpression of YY1 promotes the proliferation of AML cells, while the depletion of METTL3 reduces the proliferation-promoting effect of the cells. In addition, we found that overexpressing YY1 while silencing METTL3 could significantly reduce the promoting effect of YY1 on proliferation (Fig. 2C, D). Furthermore, we applied CCK-8 and colony formation assays to detect the mid- and long-term proliferation ability of AML cell lines with consistent experimental results (Fig. 2E–H). At the same time, we found that YY1 also rescued cell proliferation after METTL3 knockdown, suggesting that although METTL3 expression is an important factor in the regulation of AML cell proliferation by YY1, it is not the only factor.

In addition, to further determine whether METTL3 is an important functional downstream target of YY1 in AML cells, we performed METTL3 overexpression after YY1 was knocked down and assessed cell proliferation using EdU, CCK-8, and colony formation assays. The results showed that YY1 knockdown significantly reduced the proliferation of AML cells, while METTL3 overexpression rescued the reduced proliferation effect caused by YY1 depletion (Fig. S2A–F).

### HDACi treatment disrupts the binding of YY1 and HDAC1/3 and inhibits METTL3 expression and AML cell proliferation

**Fig. 3:**
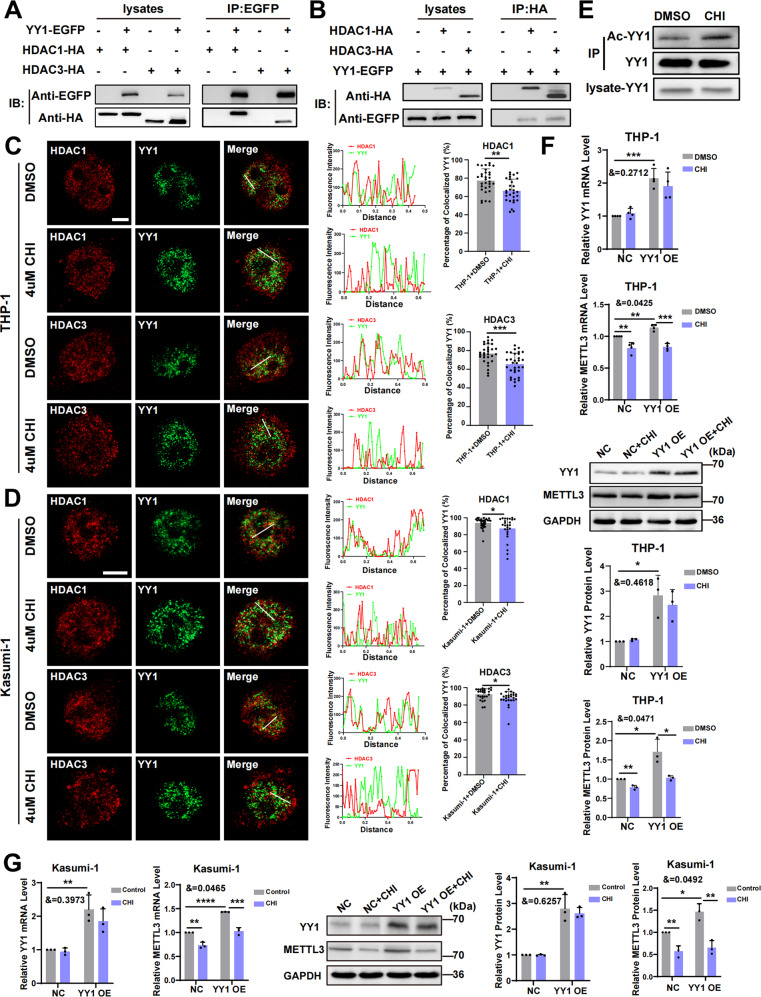
HDACi disrupts the binding of YY1 and HDAC1/3 and inhibits METTL3 expression. **A**, **B** Coimmunoprecipitation (Co-IP) assays for association of HDAC1/3 with YY1. **A** Co-transfected YY1-EGFP with HDAC1/3-HA, the cell extracts were prepared and precipitated with anti-EGFP beads, and detected by using immunoblotting with anti-EGFP and anti-HA antibodies. **B** Co-transfected HDAC1/3-HA with YY1-EGFP, the cell extracts were prepared and precipitated with anti-HA beads, and detected by using immunoblotting with anti-HA and anti-EGFP antibodies (*n* = 3). **C**, **D** Immunofluorescence colocalization of YY1 and HDAC1/3, THP-1 cells (**C**) and Kasumi-1 (**D**) cells were transfected with HDAC1/3-HA, with or without 4 μM CHI treatment for 1 h. YY1 was immunostained in green and HDAC1/3 in red. Line scans of colocalization images are depicted by white line with quantification shown at middle. The percentage of colocalized YY1 were shown at right. Scale bars, 5 μm. *P < 0.05; **P < 0.01; ***P < 0.001, *t*-test. **E** HEK293T cell extracts were prepared and precipitated with anti-YY1 antibody, and detected by using immunoblotting with anti-YY1 and pan-acetylated antibodies (*n* = 3). **F**, **G** q-PCR or western blotting analysis of YY1 and METTL3 expression at mRNA or protein level of THP-1 cells (**F**) or Kasumi-1 cells (**G**) with or without 4 μM CHI treatment for 24 or 48 h. *P < 0.05; **P < 0.01; ***P < 0.001; ****P < 0.0001, &= interaction effect, two-way ANOVA.

YY1 was reported to interact with many proteins and cofactors [20] and is regulated by acetylation and deacetylation [39]. To prove whether the transcriptional regulation of METTL3 by YY1 was related to HDACs, we performed coimmunoprecipitation experiments, which showed that HDAC1 or HDAC3 could be precipitated by YY1 (Fig. 3A). To further validate the interaction between YY1 and HDAC1 or HDAC3, coimmunoprecipitation was also performed in HEK-293T cells using anti-HA beads, and YY1 could be precipitated by HDAC1 or HDAC3 (Fig. 3B). These results supported the existence of physical interactions between YY1 and HDAC1 or HDAC3. To further analyze the function of YY1 and HDCA1 or HDAC3 in cells, confocal immunofluorescence was performed to detect the location of YY1 and HDAC1 or HDAC3 (Fig. 3C, D). This result shows that YY1 colocalized with HDAC1 or HDAC3, and compared with the DMSO-treated group, the colocalization of YY1 with HDAC1/HDAC3 was decreased after 4 μM CHI treatment for 1 h (Fig. 3C, D). These results indicated that YY1 could interact with HDAC1 or HDAC3 to form a molecular complex. To determine whether HDAC inhibitor treatment affected the acetylation of YY1, we performed immunoprecipitation assay with anti-YY1 antibody and detected its acetylation levels by pan-acetylated antibody. The results showed that the acetylation levels of YY1 was increased significantly when the cells were treated by HDAC inhibitor (Fig. 3E). To further assess the role of HDACs in the transcriptional regulation of METTL3 by YY1, THP-1, and Kasumi-1 cells were treated with CHI, while DMSO was used as a control. Using PCR and western blotting, we found that CHI could significantly reduce the expression of METTL3 in THP-1 and Kasumi-1 cells and could reverse the increase in METTL3 expression caused by YY1 overexpression (Fig. 3F, G). All these results suggested that CHI regulates YY1 and inhibits its transcriptional regulation of METTL3.

**Fig. 4:**
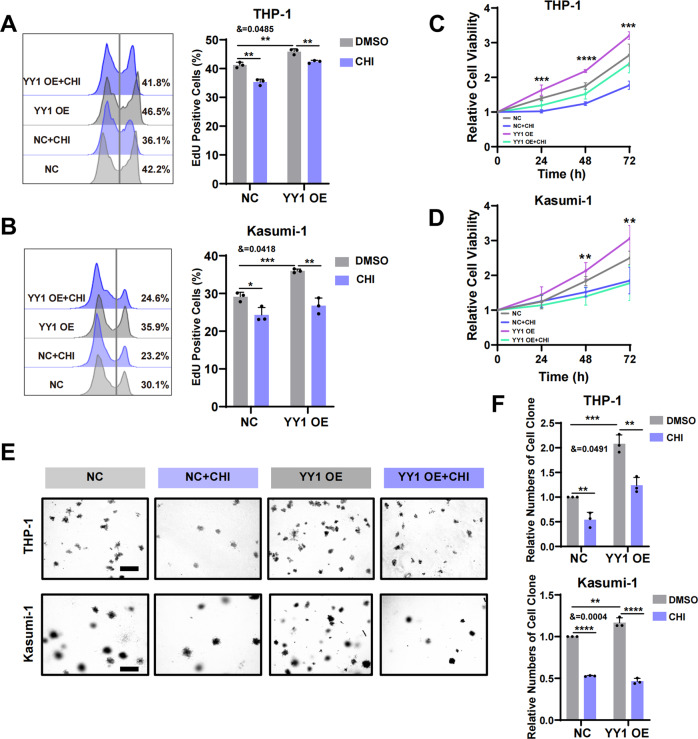
HDACi inhibits AML cells proliferation through METTL3. **A**, **B** The proliferative activity of THP-1 cells (**A**) and Kasumi-1 cells (**B**) with or without 4 μM CHI treatment for 24 h measured by flow cytometry EdU assay. *P < 0.05; **P < 0.01; ***P < 0.001, *t*-test. &= interaction effect, two-way ANOVA. **C**, **D** The proliferative activity of THP-1 cells (**C**) and Kasumi-1 cells (**D**) measured by CCK-8 assay. **P < 0.01; ***P < 0.001; ****P < 0.0001, one-way ANOVA. **E** The number of cell clone formed assessed by colony formation assay. Scale bars, 500 μm. **F** Statistical chart of colony formation experiment. **P < 0.01; ***P < 0.001; ****P < 0.0001, &= interaction effect, two-way ANOVA.

To further understand the effect of CHI on AML cell proliferation by regulating YY1, we treated THP-1 or Kasumi-1 cells with 4 μM CHI for 24 h. Flow cytometric EdU assays showed that CHI could significantly inhibit the proliferation of AML cells and reverse the accelerated cell proliferation caused by overexpression of YY1 (Fig. 4A, B). Next, we performed CCK-8 analysis to examine the effect of CHI treatment on cell proliferation at different times. The results showed that the longer the duration of CHI action was, the more significant the inhibition of AML cell proliferation was (Fig. 4C, D). The colony formation experiment showed the same result (Fig. 4E, F). In conclusion, CHI can inhibit the proliferation of AML cells by regulating YY1.

### YY1 undergoes moderate LLPS in an HDAC1/3-dependent model

**Fig. 5:**
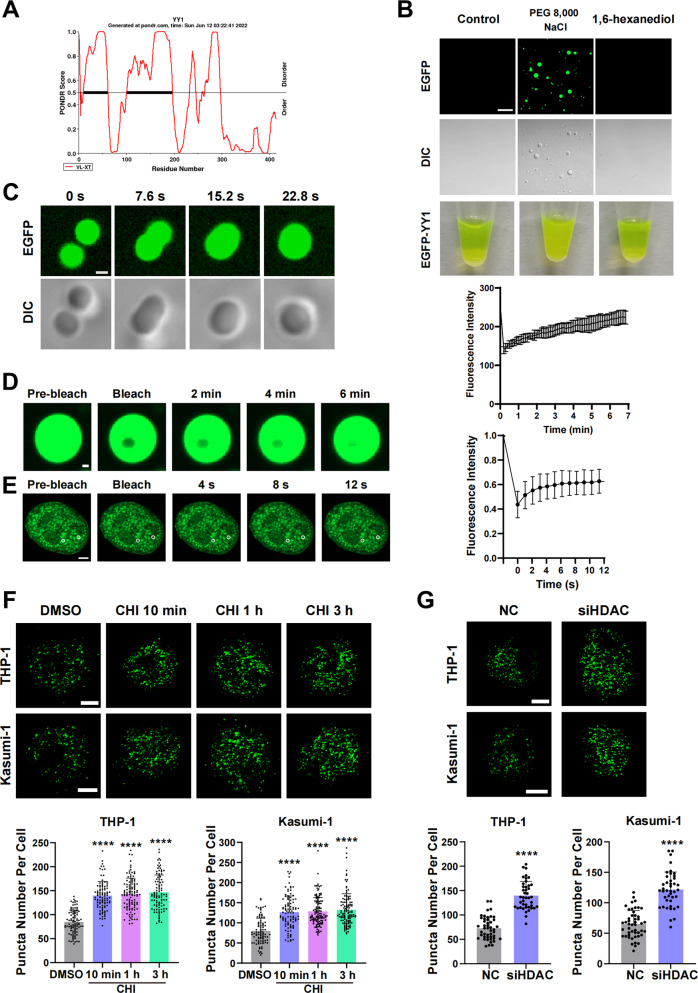
YY1 undergoes phase separation in an HDAC1/3-dependent model. **A** Disorder analysis of the YY1 amino acid isoform. The algorithms used were VLXT. The line indicates 0.5 disordered score, above which the amino acid sequence is disordered. **B** Fluorescence/DIC images and turbidity visualization of EGFP-YY1 droplet formation in a buffer containing 2 μl 4 M NaCl and 8 μl 10% PEG-8,000 (the same condition hereafter, if not specified). Scale bar, 20 μm. **C** Droplet fusion shown under fluorescent (top) and bright-field (bottom) illumination. Scale bar, 1 μm. **D** FRAP of YY1-EGFP droplets. FRAP curve is shown at right. Scale bar, 0.5 μm. **E** FRAP of YY1 in U2OS cells. FRAP curve is shown at right. Scale bar, 5 μm. **F** THP-1 and Kasumi-1 cells treated with 4 μM CHI, endogenous YY1 immunofluorescence (top), and number of fluorescent particles (bottom). ****P < 0.0001, *t*-test. Scale bars, 5 μm. **G** THP-1 and Kasumi-1 cells were transfected with si-NC or siHDAC1 and siHDAC3, endogenous YY1 immunofluorescence (top), and number of fluorescent particles (bottom). ****P < 0.0001, *t*-test. Scale bars, 5 μm.

From the immunofluorescence images in Fig. 3C, D, we found that YY1 had obvious intracellular granularity, and after HDACi treatment, the intracellular granularity was significantly increased. A study showed that YY1 could undergo LLPS, and the H and E/D clusters of the transcriptional activation domain were important elements in phase separation [36]. However, the YY1 transcriptional activation domain did not have an HDAC interaction site [39]. Meanwhile, analysis of the YY1 amino acid sequence using the PONDR website (www.pondr.com) which predicts disordered regions in proteins, revealed that YY1 also has low-complexity regions in residues 170-200 (Fig. 5A), which have been shown to have a well-defined HDAC binding site. To confirm the regulatory role of HDAC binding in this region on YY1 LLPS, we first confirmed that YY1 could undergo LLPS, validating the conclusions of the literature. Next, we induced and purified enhanced green fluorescent protein (EGFP)-YY1 in vitro. Nonionic crowder polyethylene glycol (PEG) 8000 and NaCl were added to purified EGFP-YY1; confocal imaging of EGFP-YY1 revealed the formation of micrometer-sized droplets, and turbidity in the test tube was visible to the naked eye. Moreover, droplet formation was substantially inhibited by 1,6-hexanediol (Fig. 5B). Droplet fusion and fluorescence recovery after photobleaching (FRAP) are two features of phase-separated condensates [33]. Confocal microscopy time-series imaging revealed that YY1 droplets fused into larger droplets over time, and FRAP experiments showed that YY1 recovered approximately 6 min after bleaching (Fig. 5C, D and Supplemental video.). Meanwhile, we transfected the U2OS cells with YY1-EGFP plasmid and the recovery of the fluorescence of YY1 condensed also be observed in FRAP assay (Fig. 5E). Then, we treated THP-1 and Kasumi-1 cells with CHI for 10 min/1 h/3 h. The results showed that after CHI treatment, the number of intracellular granules increased significantly (Fig. 5F). We then interfered with HDAC1 and HDAC3 expression in cells and found that the LLPS of YY1 was enhanced compared with that in the control group (Fig. 5G). These data suggested that HDACi could induce excessive LLPS of YY1, which was dependent on its separation from HDAC1/3.

### YY1 HDAC1/3 binding site mutations affects its regulation of METTL3 and AML cell proliferation

**Fig. 6:**
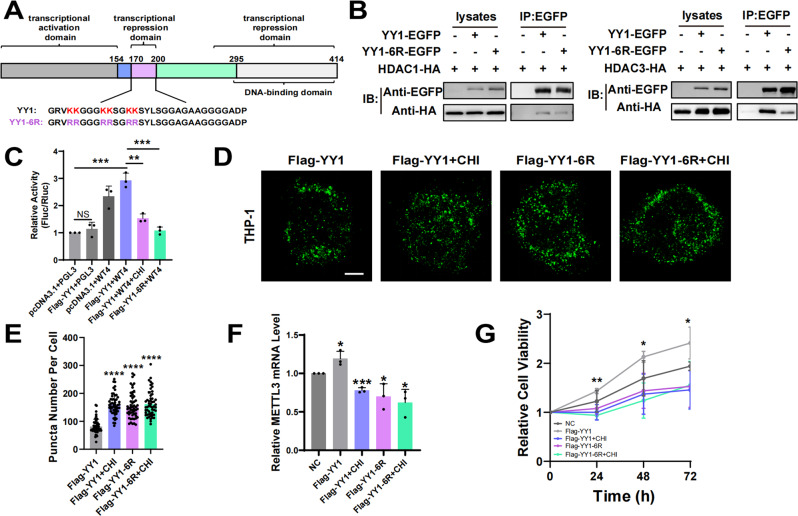
YY1 HDAC1/3 binding sites mutations affects its regulation of METTL3 and AML cell proliferation. **A** Domain structure of the YY1 protein. YY1-6R represent YY1 arginine mutant. **B** Coimmunoprecipitation (Co-IP) assays for association of HDAC1/3-HA with YY1-6R-EGFP. Co-transfected YY1-EGFP or YY1-6R-EGFP with HDAC1/3-HA, the cell extracts were prepared and precipitated with anti-EGFP beads, and detected by using immunoblotting with anti-EGFP and anti-HA antibodies (*n* = 3). **C** Luciferase activity in 293T cells co-transfected with the wide type YY1 or Flag-YY1-6R and the luciferase reporter vectors, with or without CHI treatment for 24 h, assessed by luciferase reporter assay. **P < 0.01; ***P < 0.001; NS = nonsignificant, *t*-test. **D** THP-1 cells were transfected with Flag-YY1 or Flag-YY1-6R mutant plasmids, with or without 4 μM CHI treatment for 1 h, and performed immunofluorescence analysis with anti-Flag antibody. **E** The number of fluorescent particles of figure (**D**). ****P < 0.0001, *t*-test. Scale bar, 5 μm. **F** THP-1 cells were transfected with Flag-YY1 or Flag-YY1-6R mutant plasmids, with or without 4 μM CHI treatment for 24 h, and detected the expression of METTL3 by qPCR. *P < 0.05; ***P < 0.001, *t*-test. **G** The proliferation ability of THP-1 cells transfected with Flag-YY1 or Flag-YY1-6R mutant plasmids, with or without 4 μM CHI treatment, and detected by CCK-8 assay. *P < 0.05; **P < 0.01, one-way ANOVA.

Six lysines arranged in pairs within YY1 residues 170 to 200 have been reported as HDAC binding sites of YY1 [40]. To monitor the effect of HDAC1/3 on LLPS, we constructed a mutant YY1 containing six amino acid substitutions (K-R) in the central HDAC binding domain [33] (Fig. 6A). To examine the effect of the mutant on the binding of YY1 to HDAC1/3, we cotransfected HEK293T cells with the YY1-EGFP/YY1-6R-EGFP construct with HDAC1/3-HA plasmid, followed by coimmunoprecipitation analysis. The results showed that the binding of YY1-6R to HDAC1/3 was reduced compared with that of wild-type YY1 (Fig. 6B). We then performed dual luciferase report assay and found that HDAC inhibition or mutated YY1 reduced the ability for YY1 to bind to the METTL3 promoter and reduced promoter luciferase activity (Fig. 6C). Next, we transfected wild-type or YY1 mutant plasmids into THP-1 cells and performed immunofluorescence analysis with an anti-Flag antibody. The results showed that the LLPS of the YY1 mutant was improved (Fig. 6D, E). To explore whether the mutant could affect the regulation of METTL3 by YY1, we transfected YY1 wild-type and mutant YY1 plasmid into THP-1 cells and detected the expression of METTL3 by RT-qPCR (Fig. 6F). At the same time, the effect of the mutant on the proliferation ability of THP-1 cells was detected by CCK-8 assay (Fig. 6G). Meanwhile, HDACi had no influence on the LLPS of YY1-6R, the METTL3 expression and the cell proliferation, compared with that in YY1-6R group. All the results indicated that the failure of YY1 binding with HDAC affected its regulation of METTL3, which in turn affected the proliferation of AML cells.

## Discussion

In this study, we proved that YY1 underwent moderate LLPS in an HDAC1/3-dependent manner to promote METTL3 expression and ultimately AML proliferation. Then, HDAC1/3 was confirmed as a key factor in regulating the LLPS of YY1, and exploiting this mechanism via HDACi treatment is a feasible strategy for AML treatment. Our research has the following innovations.

First, we clarified the specific activator of METTL3 upregulation in AML cells. METTL3 has been demonstrated to play a variety of biological roles in various diseases, including AML [41–46], and a newly reported small-molecule METTL3 inhibitor reduced AML cell growth and promoted AML cell differentiation and apoptosis [8]. However, METTL3 has many basic functions to ensure the survival of animals [47]; for example, METTL3 plays an active role in mammalian spermatogenesis. Loss of m^6^A methylation by METTL3 deficiency disrupted spermatogonial stem cell (SSC)/progenitor cell homeostasis [48]. METTL3 is a key regulator of skeletal muscle differentiation [49]. Clarifying the regulatory mechanism of abnormal METTL3 expression is of great significance for precise targeting. Under different pathological conditions, cells can regulate the abnormal expression of METTL3 through different expression regulators. Clarifying the mechanism of the abnormal expression of MELLT3 in AML is crucial for targeting and interfering with METTL3 dysregulation in AML cells. Our results revealed that YY1 induced METTL3 expression by binding to the promoter regions of METTL3 and that HDACi treatment significantly reduce the binding of YY1 to HDAC1/3, thereby reducing the expression of METTL3. Our study revealed that YY1 is a new regulator of METTL3 expression and clarified the specific mechanism by which HDACi inhibits YY1 to regulate METTL3 expression. This may provide a new target for the treatment of AML. In addition, it provides additional strong evidence for the application of HDACis in the treatment of AML.

Second, we delineated the mechanism of the fine-tuning of YY1 function from the perspective of LLPS degree. YY1 is an important transcription factor for zinc-finger proteins [20], which can play both transcriptional activation and transcriptional repression functions [21, 50]. YY1 has been reported to be abnormally expressed in a variety of tumors and is critical for tumor progression [51, 52]. It is worth noting that the mechanism of positive and negative regulation of YY1 is still relatively obscure. Research has shown that IDRs in the transcriptional activation domain of YY1 can promote the LLPS of YY1 [36]. However, our study found that the IDRs of the transcriptional repression domain of YY1 could regulate LLPS in an HDAC1/3-dependent manner. This seemingly contradictory result seems to explain the mechanism of positive and negative regulation of YY1 better than previous theories. Phase separation is a state of dynamic equilibrium [53] and “moderate” LLPS has been proved important for many biological function, for example, WNK1 undergoes hypertonic pressure-induced dynamic phase separation to restore cell volume [54]; HSPB1 regulates the reversible TDP-43 phase separation from liquid to gel/solid [55]. In our study, broadly defined YY1 underwent LLPS, promoting the METTL3 expression and the proliferation of AML cells. This natural YY1 LLPS state would be mild LLPS. However, after treatment with HDACi or mutation of the HDAC binding site, YY1 dissociated from HDAC1/3 and a significant increase in intracellular granules was found compared to the wild-type group. The failure of YY1 to bind to HDAC resulted in its excessive LLPS and failed to enhance METTL3 expression. For the reasons, this may be because the excessive LLPS of YY1 leads to a certain degree of steric hindrance, thereby inhibiting its promoting effect on METTL3 expression. In addition, in phase-separated droplets, YY1 may interact with more cofactors to promote or reverse the original transcriptional regulation effects. We found that the function of “yin” or “yang” depends on the degree of its LLPS, which provides a new way of thinking to understand the role of YY1. However, whether the excessive phase separation state of YY1 affects the binding of other cofactors or the levels of epigenetic modifications of YY1 still needs to be further explored.

In conclusion, we found that in AML cells, YY1 regulates the abnormal expression of METTL3 by binding to its promoter region and ultimately regulates the proliferation of AML cells. We also confirmed that the LLPS of YY1 is involved in the regulation of METTL3 expression, and confirmed that this regulatory effect can be suppressed regulated by HDACi treatment. Therefore, our study clarified the importance of the LLPS degree of YY1 on the proliferation of AML cells, provides a new therapeutic target for the high expression of METTL3 in AML, and provides new theoretical support for the clinical application of HDACis.

## Supplementary information

Supplemental Data 1

FRAP video

Supplementary Figures and Figure Legends

Full and uncropped western blots

Reproducibility checklist

## Data Availability

The experimental data sets generated and/or analyzed during the current study are available from the corresponding author upon reasonable request.
