# Supplementary material for: HDAC1/3-dependent moderate liquid–liquid phase separation of YY1 promotes METTL3 expression and AML cell proliferation
Source: Cell Death Dis. 2022 Nov 24;13(11):992. doi: 10.1038/s41419-022-05435-y (PMC9691727; doi:10.1038/s41419-022-05435-y)
Supplement: Supplementary file 1 — Supplemental Data 1 [file 41419_2022_5435_MOESM1_ESM.doc]

**Supplementary Table 1.** List of primers.

| Name | Sequence | Note |
| --- | --- | --- |
| YY1-F | AGCAGAAGCAGGTGCAGATCAA | qPCR of human genes |
| YY1-R | CTGCCAGTTGTTTGGGATCT |
| YY1-F-2 | TCAGATCCCAAACAACTGGCA |
| YY1-R-2 | GGCCGAGTTATCCCTGAACA |
| METTL3-F | TGATGCTGATCGACCCTGTC |
| METTL3-R | CTTGGCGTGTGGTCTTTGC |
| GAPDH-F | GCACCGTCAAGGCTGAGAAC |
| GAPDH-R | TGGTGAAGACGCCAGTGGA |
| Primer 1-F | AAAAGTCTATGCCCATGCTACCT | CHIP-qPCR |
| Primer 1-R | GCCATACGCTGTTGTCCAAAAA |
| Primer 2-F | ACAAACAAAAGGCCAAGCCG |
| Primer 2-R | TGCCACCATGCCCAGTTAAT |
| Primer 3-F | TGAGGCAGGAGAATCGCT |
| Primer 3-R | TACAAGGTTGGTGGTGGTGG |
| Primer 4-F | CACTTGAACCCGGGAGGT |
| Primer 4-R | CGGAGAGGAGTCCTGAGAGT |

**Supplementary Table 2.** List of antibodies.

| Antibodies | Source | Identifier |
| --- | --- | --- |
| Anti-YY1 Antibody | abcam | Cat# ab38422 |
| Anti-YY1 Antibody | Cell Signaling Technology | Cat# 63227S |
| Anti-YY1 Antibody | Santa Cruz Biotechnology | Cat# sc7341 |
| METTL3/MT-A70 Antibody | Bethyl | Cat# A301-568A |
| Acetylated-Lysine Antibody | Cell Signaling Technology | Cat# 9441 |
| GAPDH Antibody | Abways Technology | Cat# AB0037 |
| GFP Polyclonal Antibody | Thermo Fisher Scientific | Cat# A-11122 |
| HA Tag Antibody | Proteintech | Cat# 66006-2-lg |
| DYKDDDDK tag Antibody | Cell Signaling Technology | Cat# 2368 |
| Horseradish Conjugated Goat Anti-Rabbit IgG (H+L) | ZSGB-BIO | Cat# ZB2301 |
| Horseradish Conjugated Goat Anti-Mouse IgG (H+L) | ZSGB-BIO | Cat# ZB2305 |
| Donkey anti-Rabbit IgG (H+L) Alexa Fluor™ 488 | Thermo Fisher Scientific | Cat# A-21206 |
| Donkey anti-Mouse IgG (H+L) Alexa Fluor™ 594 | Thermo Fisher Scientific | Cat# A21203 |

**Supplementary Table 3.** Interference sequences.

| Oligonucleotide Names | Sequences (5'-3') |
| --- | --- |
| shYY1-1 | CGACGACTACATTGAACAAAC |
| shYY1-2 | GGGAGCAGAAGCAGGTGCAGAT |
| shMETTL3-1 | GGAGATCCTAGAGCTATTA |
| shMETTL3-2 | GCTGCACTTCAGACGAATTAT |
| siHDAC1-1 | GCAAGCAGATGCAGAGATT |
| siHDAC1-2 | CUAAUGAGCUUCCAUACAA |
| siHDAC3-1 | GAGCAACCCAGCTGAACAA |
| siHDAC3-2 | GTCCTGCATTACGGTCTCT |

**Additional file.**

**Plasmid sequence of Dual-luciferase reporter assays.**

METTL3-WT1: AAAAGTCTATG**CCCATG**CTACCTCATGCTGTGAAGATGGTTCAGTATTAATAAACTGGCTCACAGCTCCTTGACCAGATTATAAACTCTTTGAAAACAGGA**GCCATG**CCAGTCAAACTATTTTTTGTATTTTTTGGACAACAGCGTATGGC

METTL3-MUT1:

AAAAGTCTATG**CCCTAG**CTACCTCATGCTGTGAAGATGGTTCAGTATTAATAAACTGGCTCACAGCTCCTTGACCAGATTATAAACTCTTTGAAAACAGGA**GCCTAG**CCAGTCAAACTATTTTTTGTATTTTTTGGACAACAGCGTATGGC

METTL3-WT4:

CACTTGAACCCGGGAGGTGGAGGTTGCTGTGAGCCGAGATCGC**GCCATT**GCACTCCAGCCAGGGCGACAGAGTGAAACTCCGTCTCAAAAAAAAAAAAAAGAAAAAAACCCCCAAAACTGTACTATATGGTGTTGCTCTCTACGAGTGCTACTTTAACACAAGTCAACTCTCAGGACTCCTCTCCG

METTL3-MUT4:

CACTTGAACCCGGGAGGTGGAGGTTGCTGTGAGCCGAGATCGC**GCCTAT**GCACTCCAGCCAGGGCGACAGAGTGAAACTCCGTCTCAAAAAAAAAAAAAAGAAAAAAACCCCCAAAACTGTACTATATGGTGTTGCTCTCTACGAGTGCTACTTTAACACAAGTCAACTCTCAGGACTCCTCTCCG
