# Supplementary material for: HDAC1/3-dependent moderate liquid–liquid phase separation of YY1 promotes METTL3 expression and AML cell proliferation
Source: Cell Death Dis. 2022 Nov 24;13(11):992. doi: 10.1038/s41419-022-05435-y (PMC9691727; doi:10.1038/s41419-022-05435-y)
Supplement: Supplementary file 3 — Supplementary Figures and Figure Legends [file 41419_2022_5435_MOESM3_ESM.docx]

**Supplementary Figures and Figure Legends**


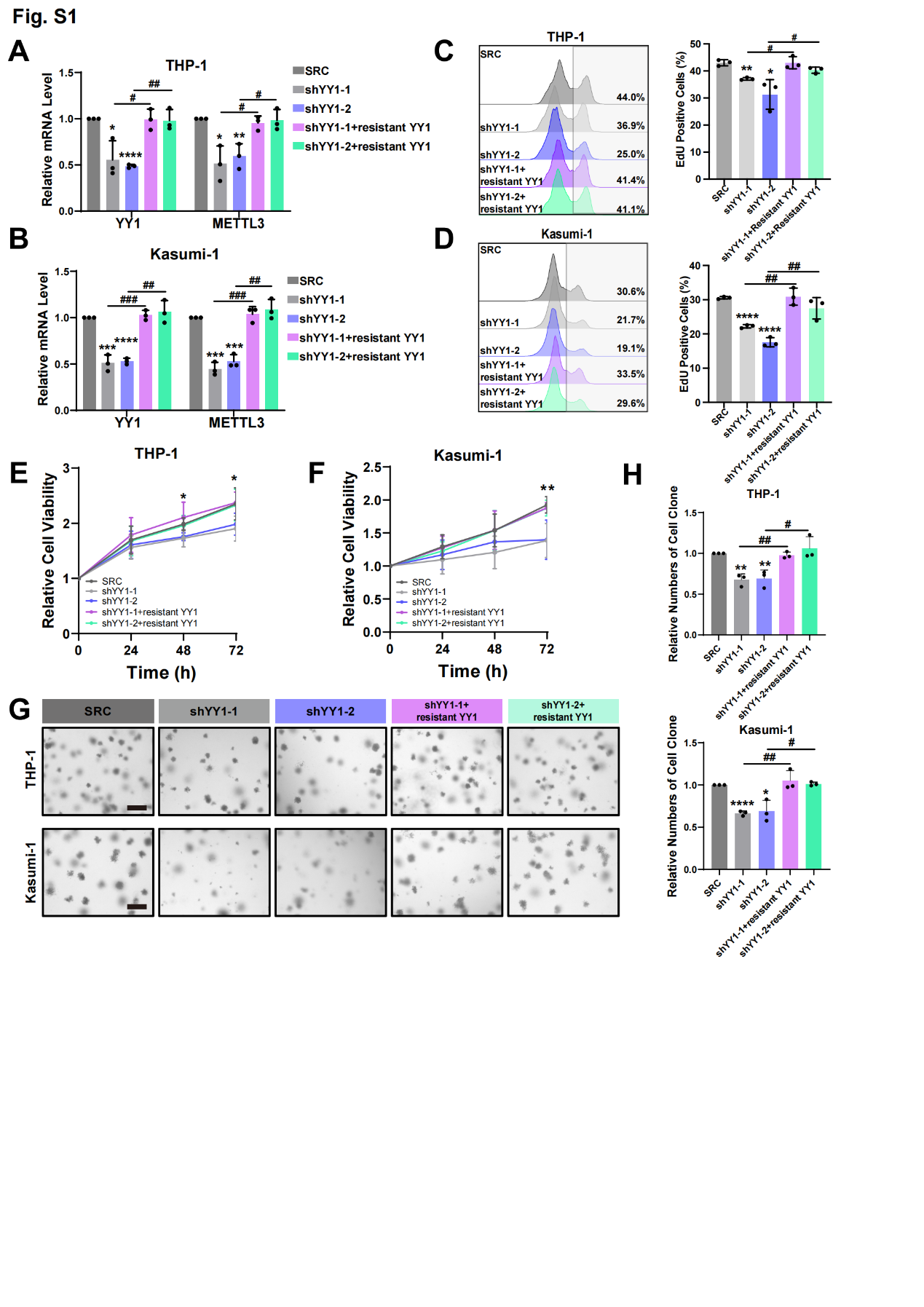


**Fig. S1 YY1 knockdown decreases the AML cell proliferation.** (A-B) qPCR analysis of the expression of YY1 and METTL3 in THP-1 cells (A) or Kasumi-1 (B) cells transfected with LV3-shYY1-1 or LV3-shYY1-2 or LV3-SRC and resistant YY1 plasmid. *Compare to the SRC group. *P < 0.05; **P < 0.01; ***P < 0.001; ****P < 0.0001; ^#^P <0.05; ^##^P <0.01; ^###^P <0.001, t-test. (C-D) The proliferative activity of THP-1 (C) and Kasumi-1 (D) cells measured by flow cytometry EdU assay. *Compare to the SRC group. *P < 0.05; **P < 0.01; ****P < 0.0001; ^#^P <0.05; ^##^P <0.01, t-test. (E-F) The proliferative activity of THP-1 cells (E) and Kasumi-1 (F) cells measured by CCK-8 assay. *P < 0.05; **P < 0.01, one-way ANOVA. (G) The number of cell clone formed assessed by colony formation assay. Scale bars, 500 μm. (H) Statistical chart of colony formation experiment. *Compare to the SRC group. *P < 0.05; **P < 0.01; ****P < 0.0001; ^#^P <0.05; ^##^P <0.01, t-test.


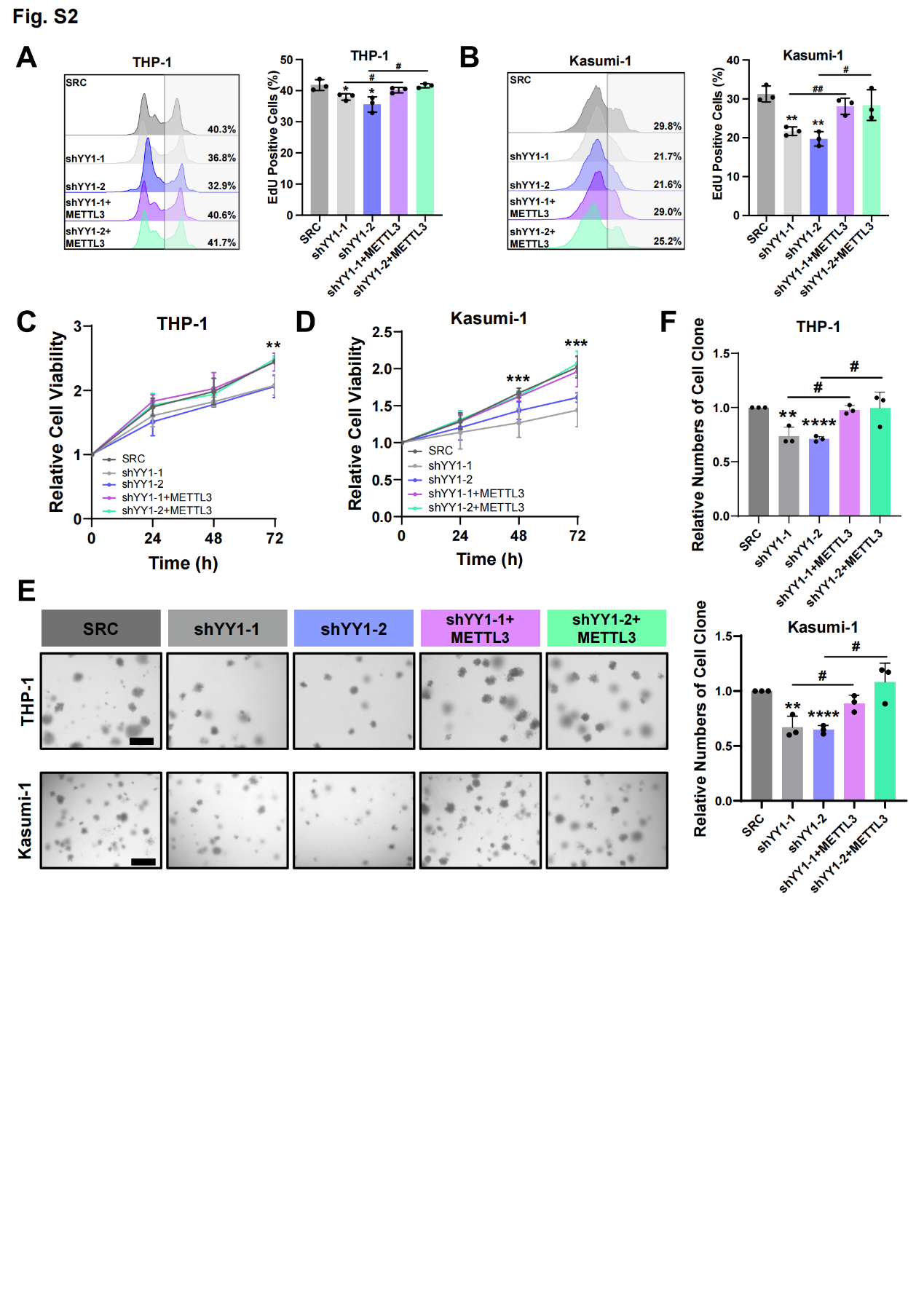


**Fig. S2 METTL3 is a critical functional downstream target of YY1 in AML cells.** (A-B) The proliferative activity of THP-1 cells (A) and Kasumi-1 (B) cells measured by flow cytometry EdU assay. *Compare to the SRC group. *P < 0.05; **P < 0.01; ^#^P <0.05; ^##^P <0.01, t-test. (C-D) The proliferative activity of THP-1 cells (C) and Kasumi-1 (D) cells measured by CCK-8 assay. **P < 0.01; ***P < 0.001, one-way ANOVA. (E) The number of cell clone formed assessed by colony formation assay. Scale bars, 500 μm. (F) Statistical chart of colony formation experiment. *Compare to the SRC group. **P < 0.01; ****P < 0.0001; ^#^P <0.05, t-test.


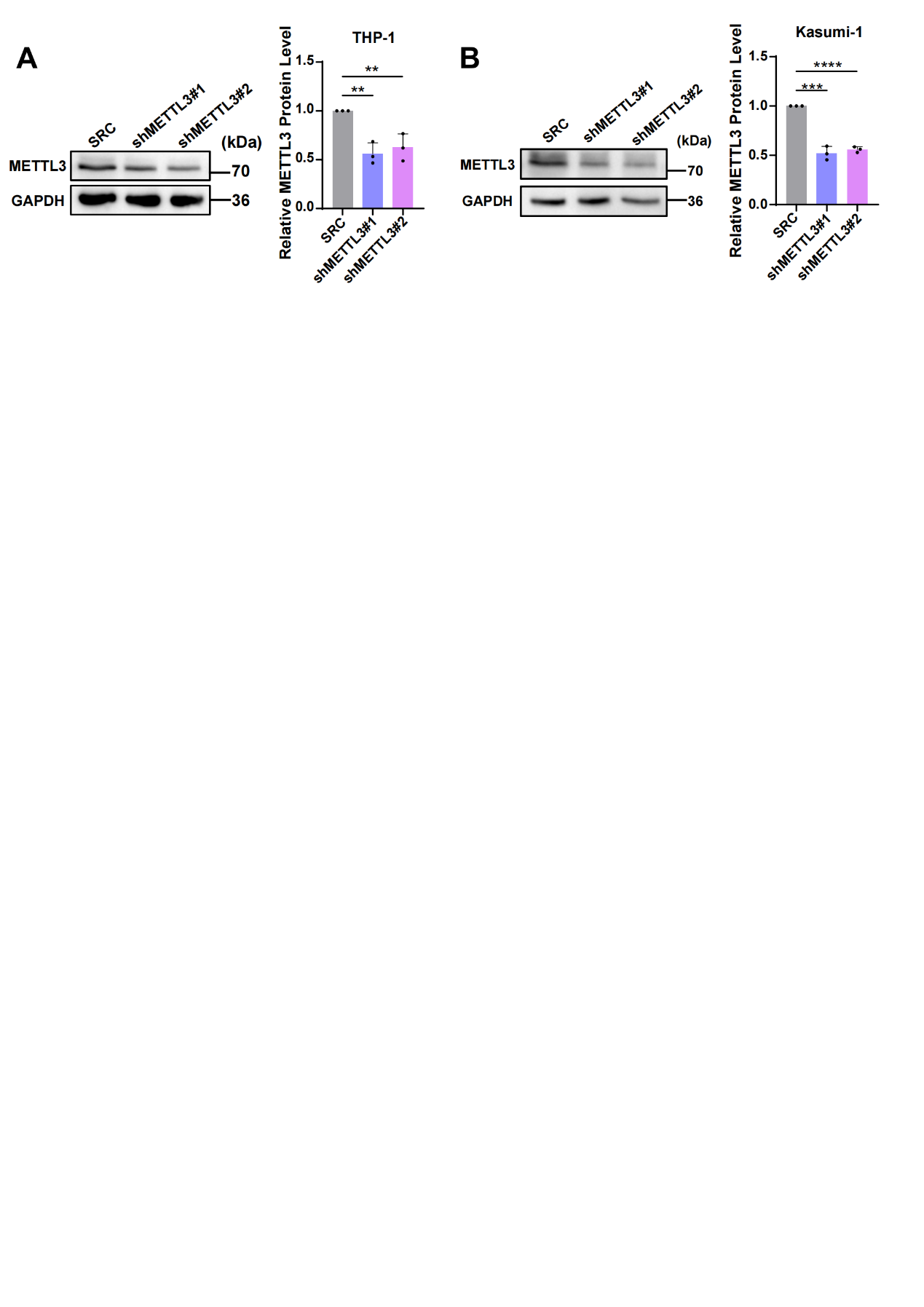


**Fig. S3 The extent of knockdown after METTL3 depletion.** (A-B) Western blotting analysis of the expression of METTL3 in THP-1 or Kasumi-1 cells that interfere METTL3. **P < 0.01; ***P < 0.001; ****P < 0.0001, t-test.
