# Supplementary material for: HDAC1/3-dependent moderate liquid–liquid phase separation of YY1 promotes METTL3 expression and AML cell proliferation
Source: Cell Death Dis. 2022 Nov 24;13(11):992. doi: 10.1038/s41419-022-05435-y (PMC9691727; doi:10.1038/s41419-022-05435-y)
Supplement: Supplementary file 4 — Full and uncropped western blots [file 41419_2022_5435_MOESM4_ESM.docx]

**Full and uncropped western blot for Figure 1 E (YY1 OE)**

Lanes 1, 2 are on the figure


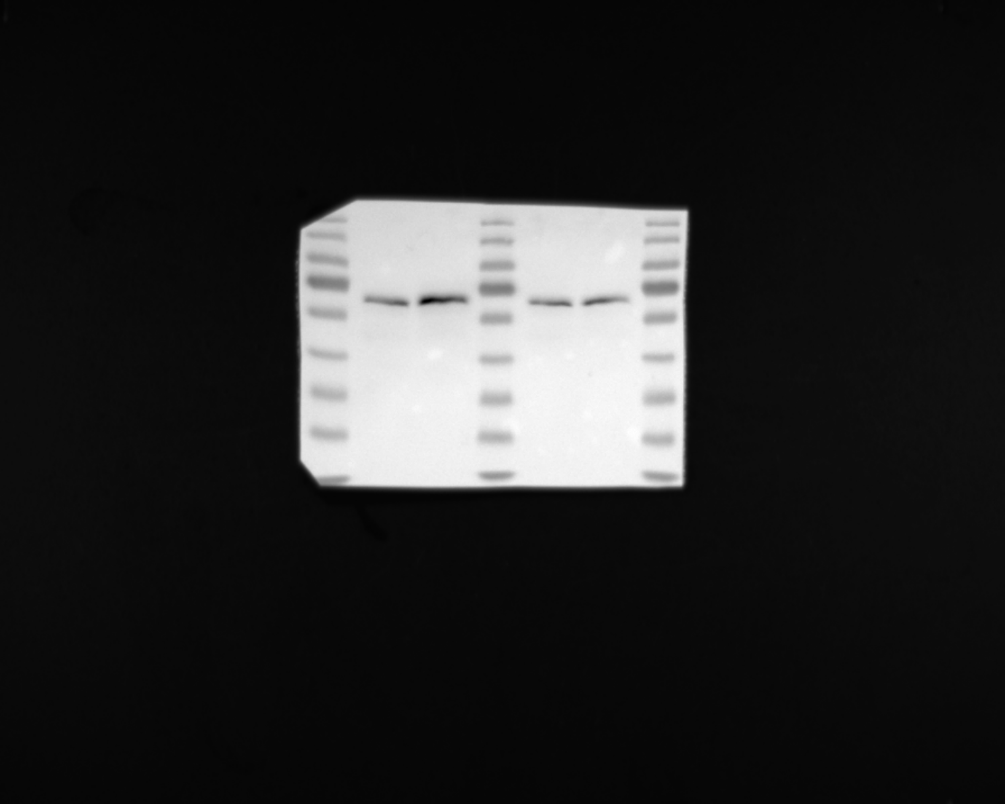

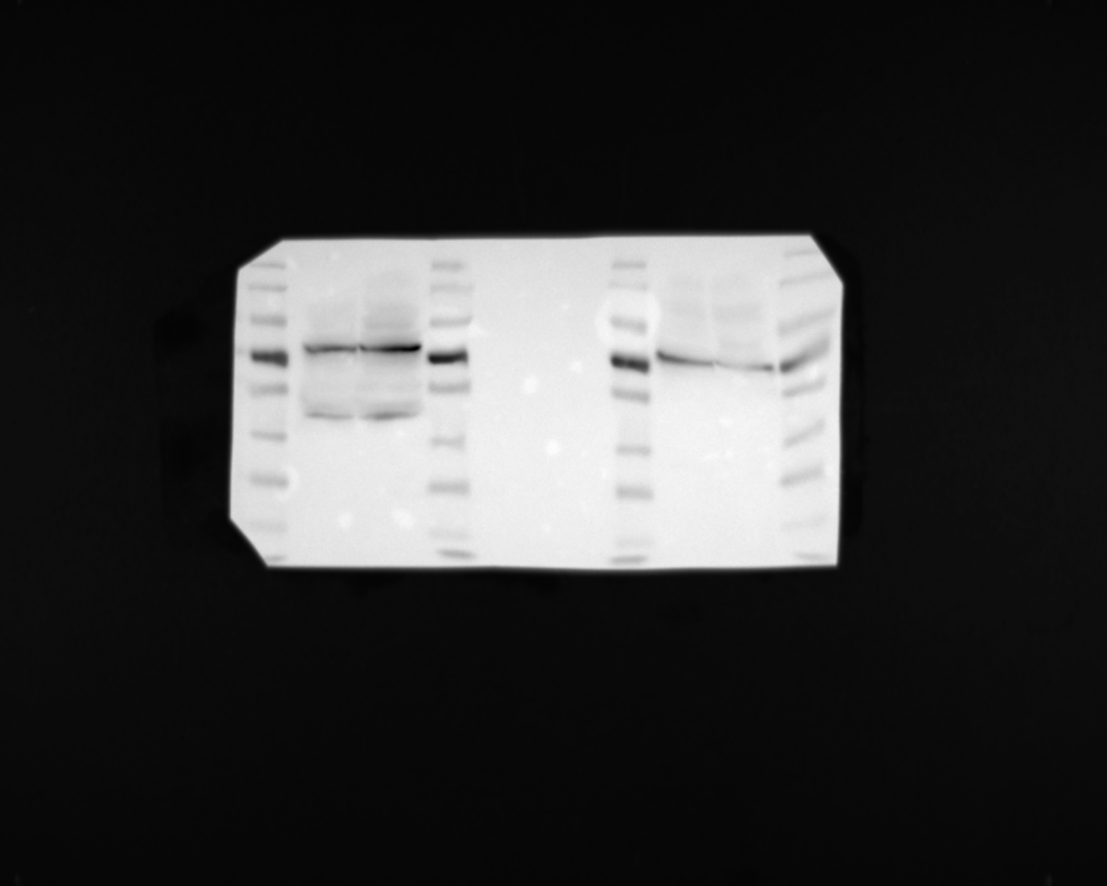


METTL3

2

1

1

2

YY1


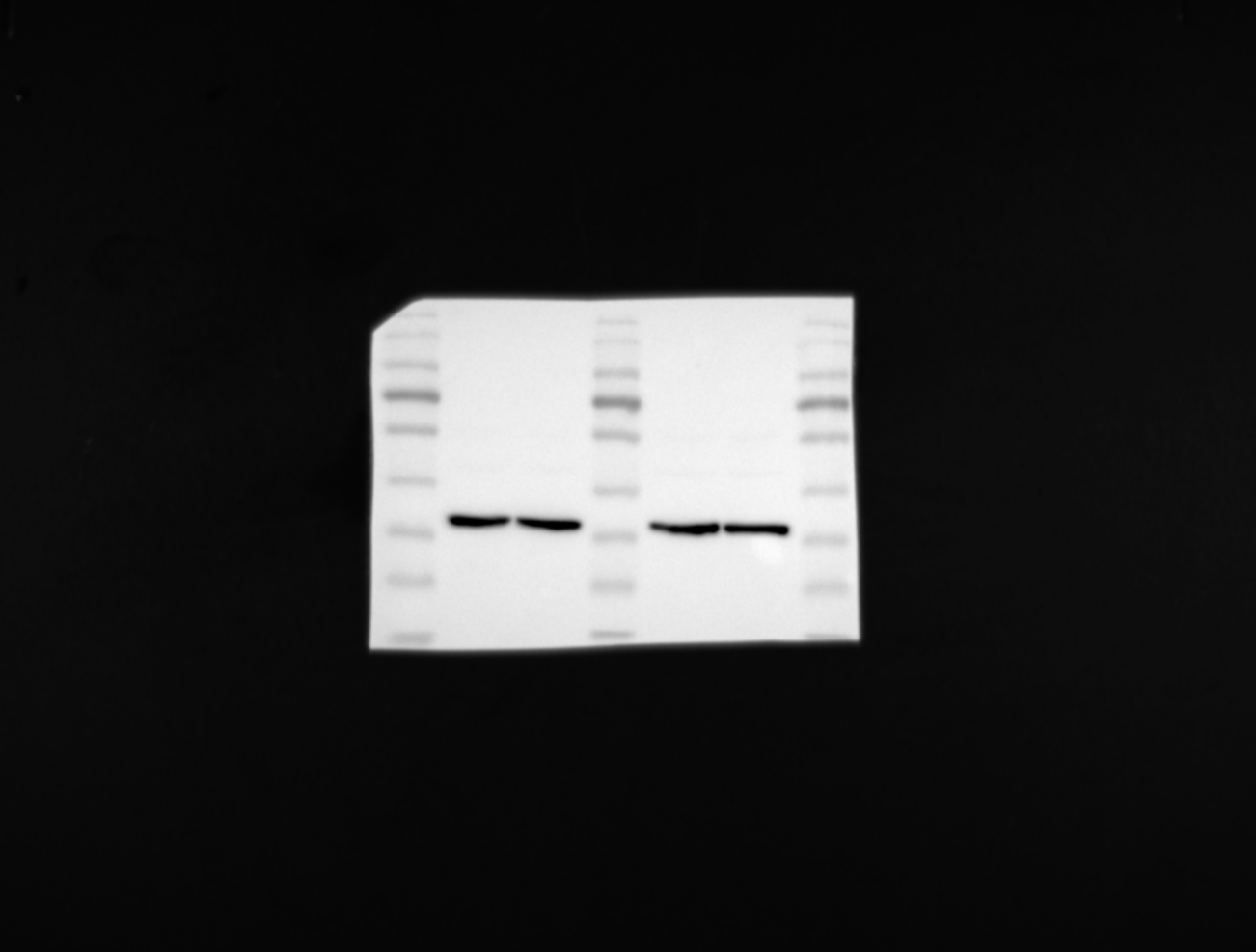


2

1

GAPDH

**Full and uncropped western blot for Figure 1 E (shYY1)**

Lanes 1, 2, 3 are on the figure


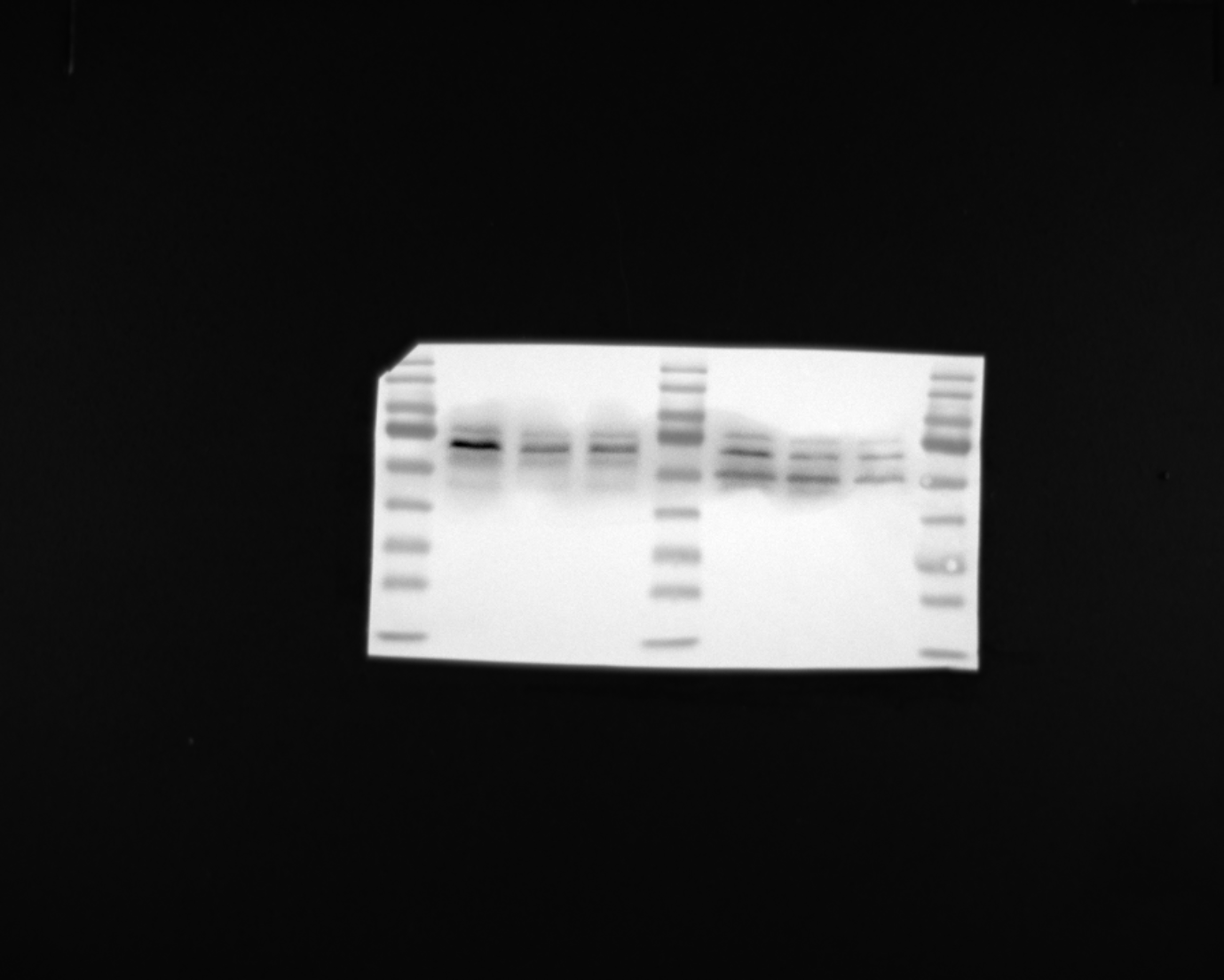

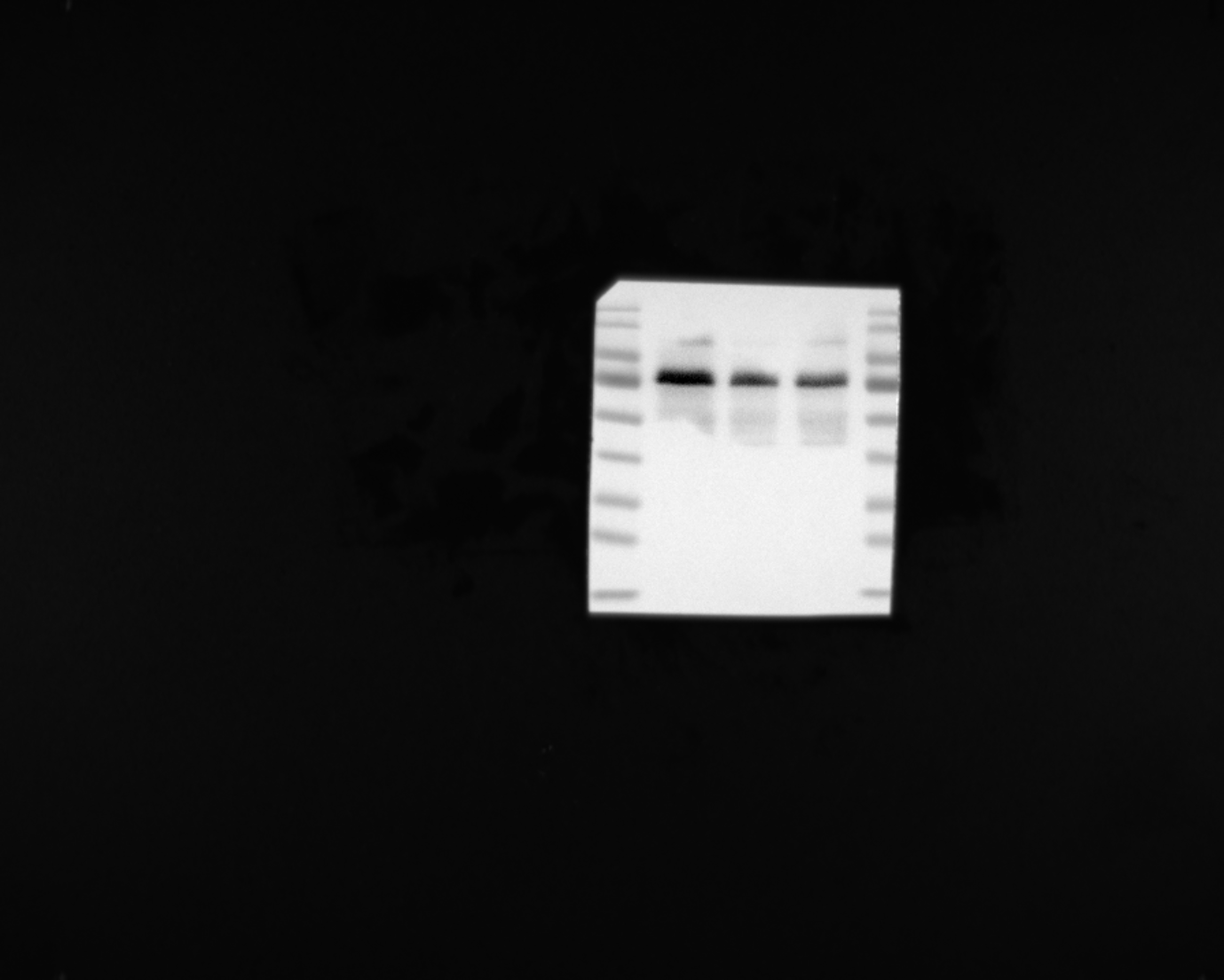


2

3

1

METTL3

1

3

2

YY1


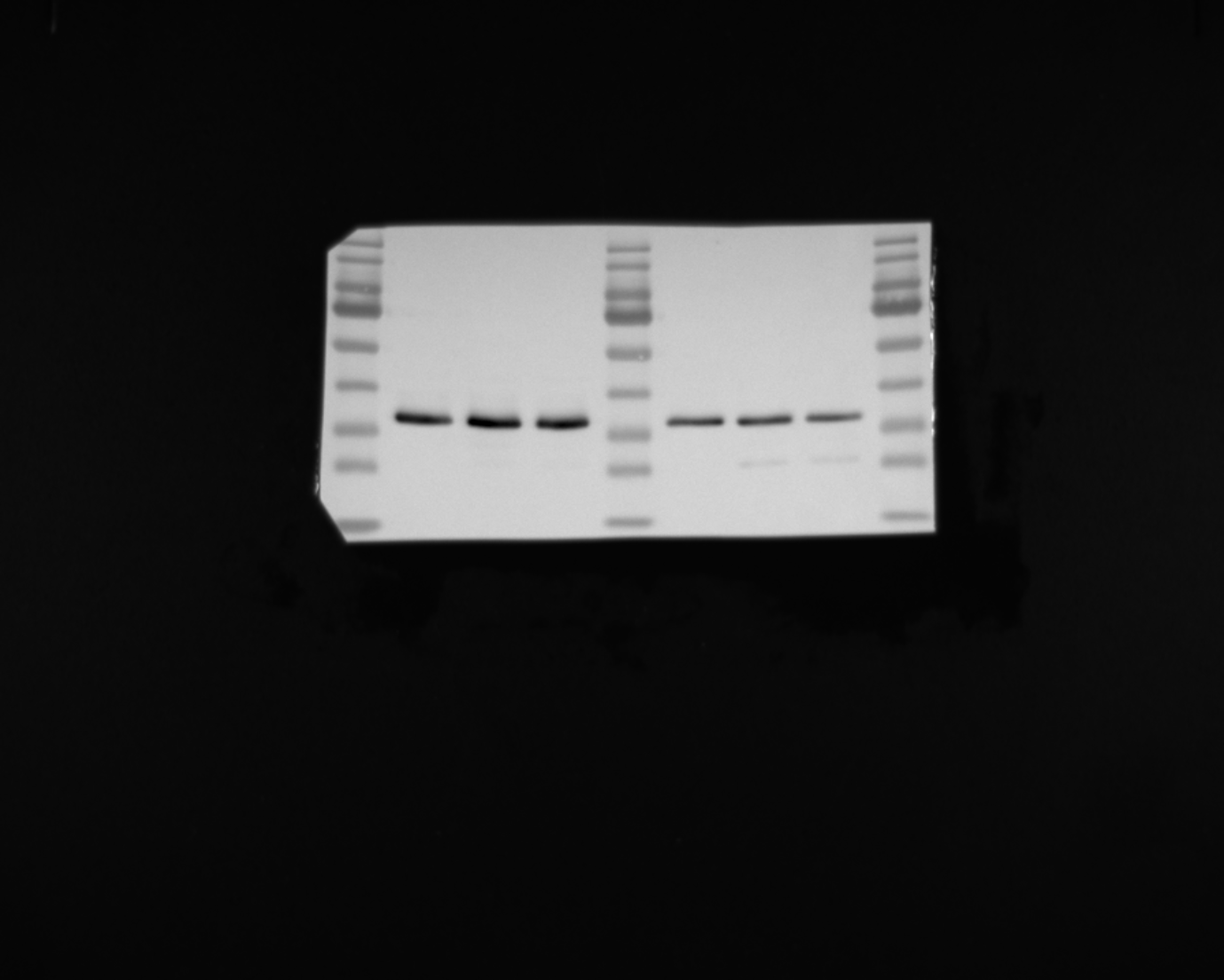


2

3

1

GAPDH

**Full and uncropped western blot for Figure 1 F (YY1 OE)**

Lanes 1, 2 are on the figure


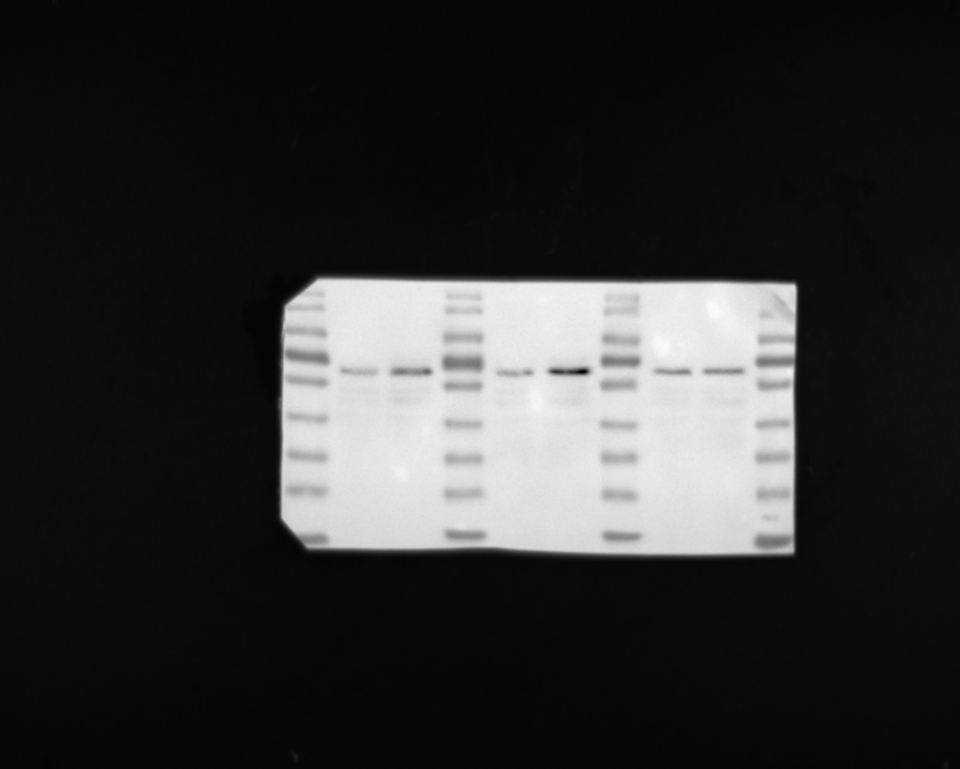

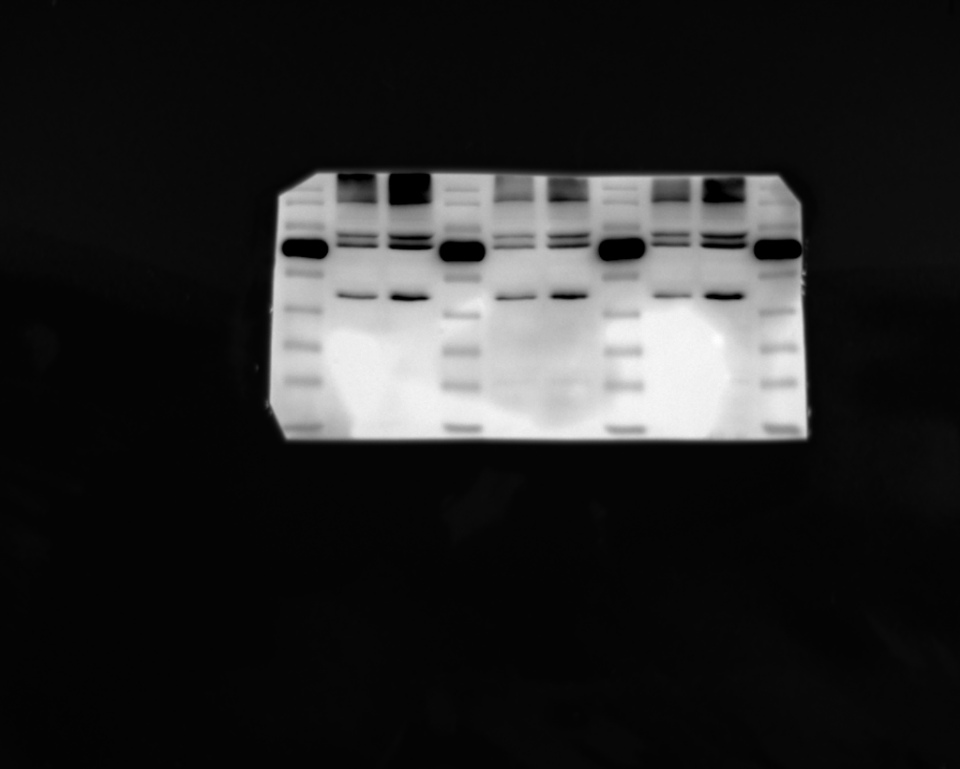


2

1

METTL3

2

1

YY1


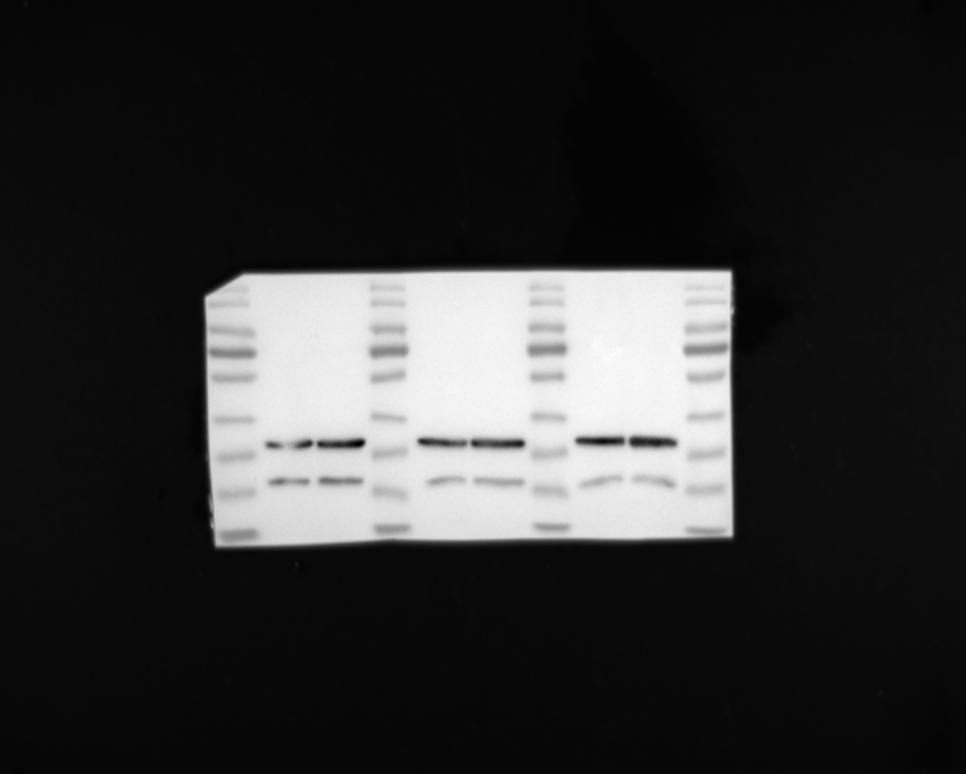


2

1

GAPDH

**Full and uncropped western blot for Figure 1 F (shYY1)**

Lanes 1, 2, 3 are on the figure


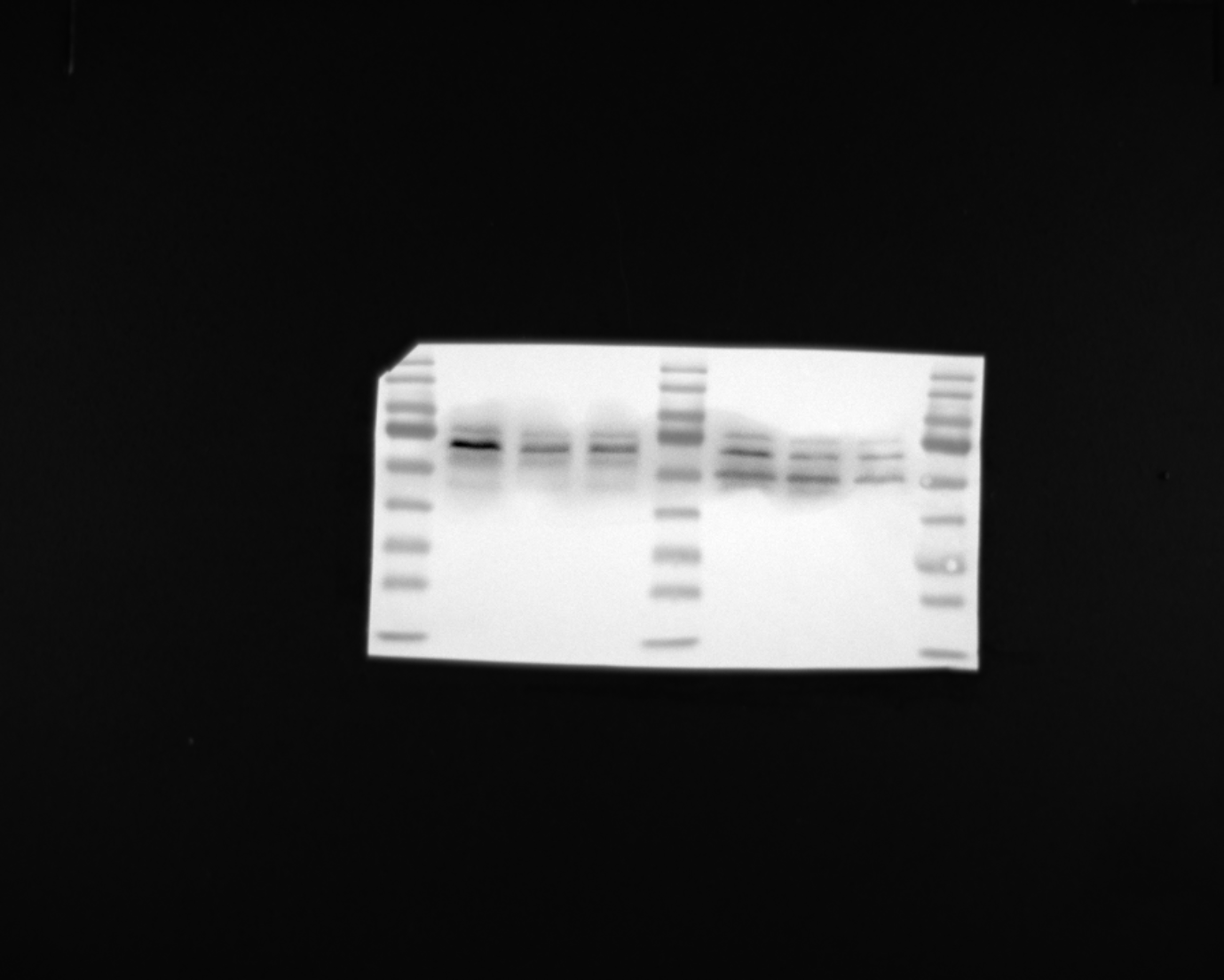

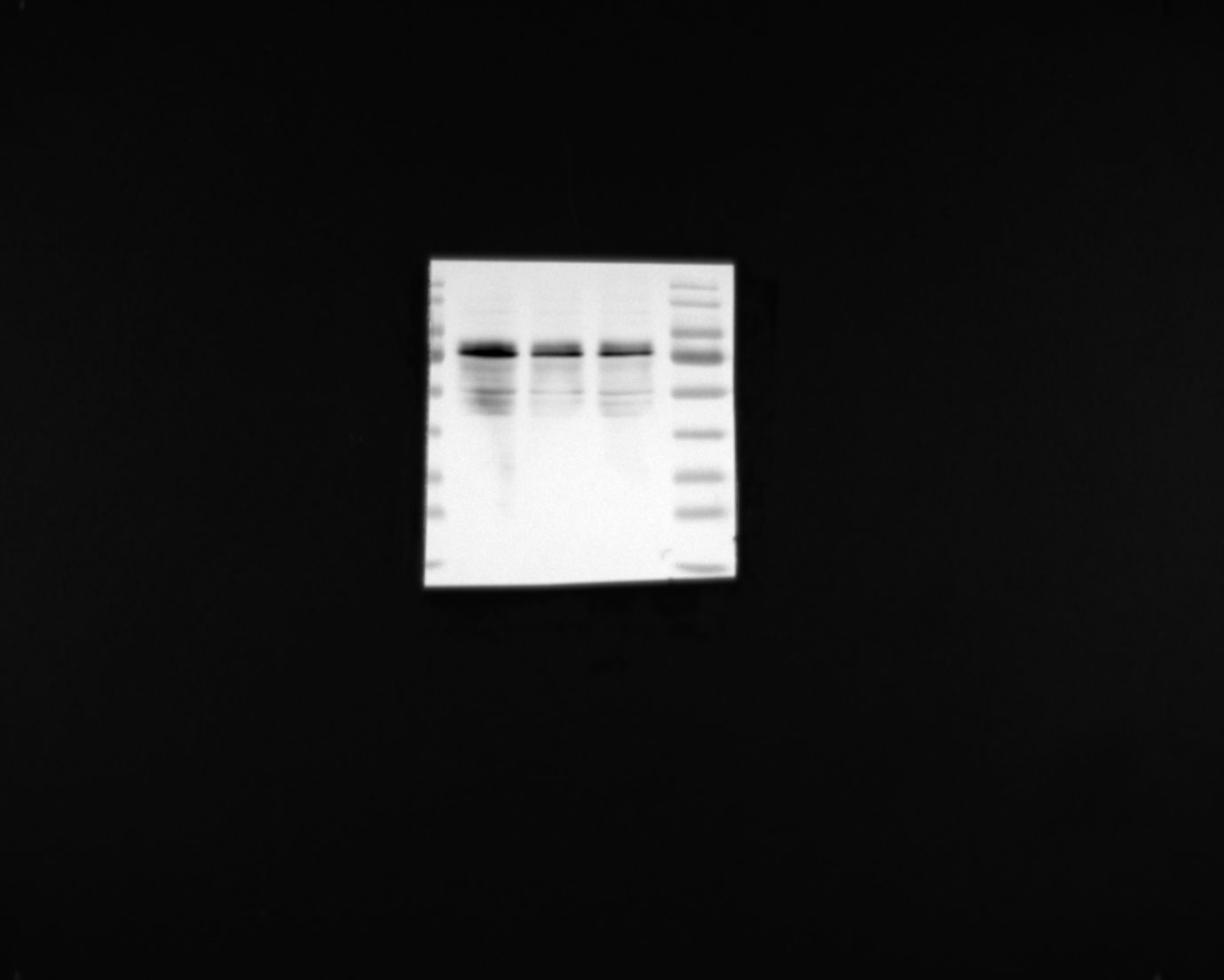


1

2

3

METTL3

1

2

3

YY1


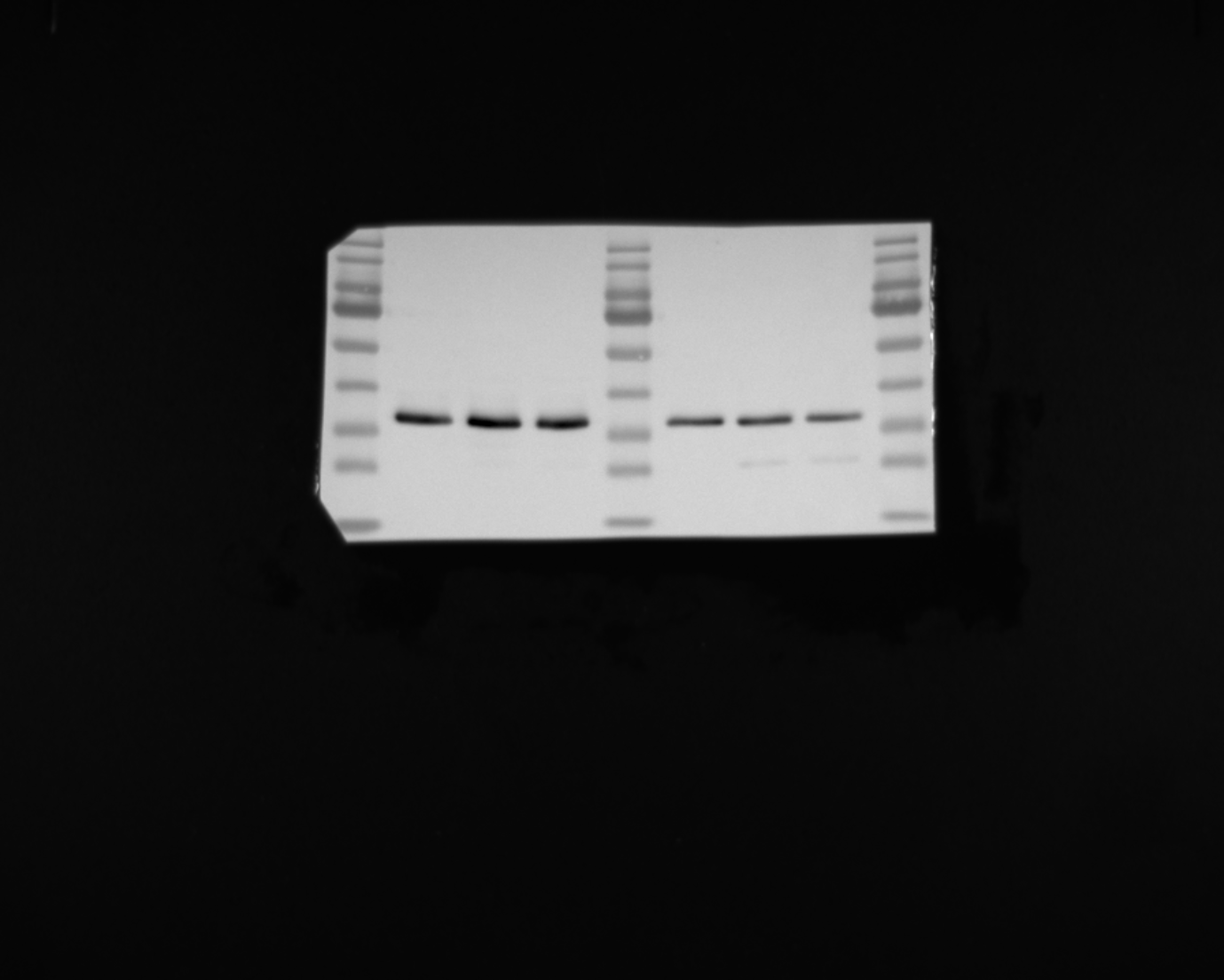


2

3

1

GAPDH

**Full and uncropped western blot for Figure 2 A**

Lanes 1, 2, 3, 4 are on the figure


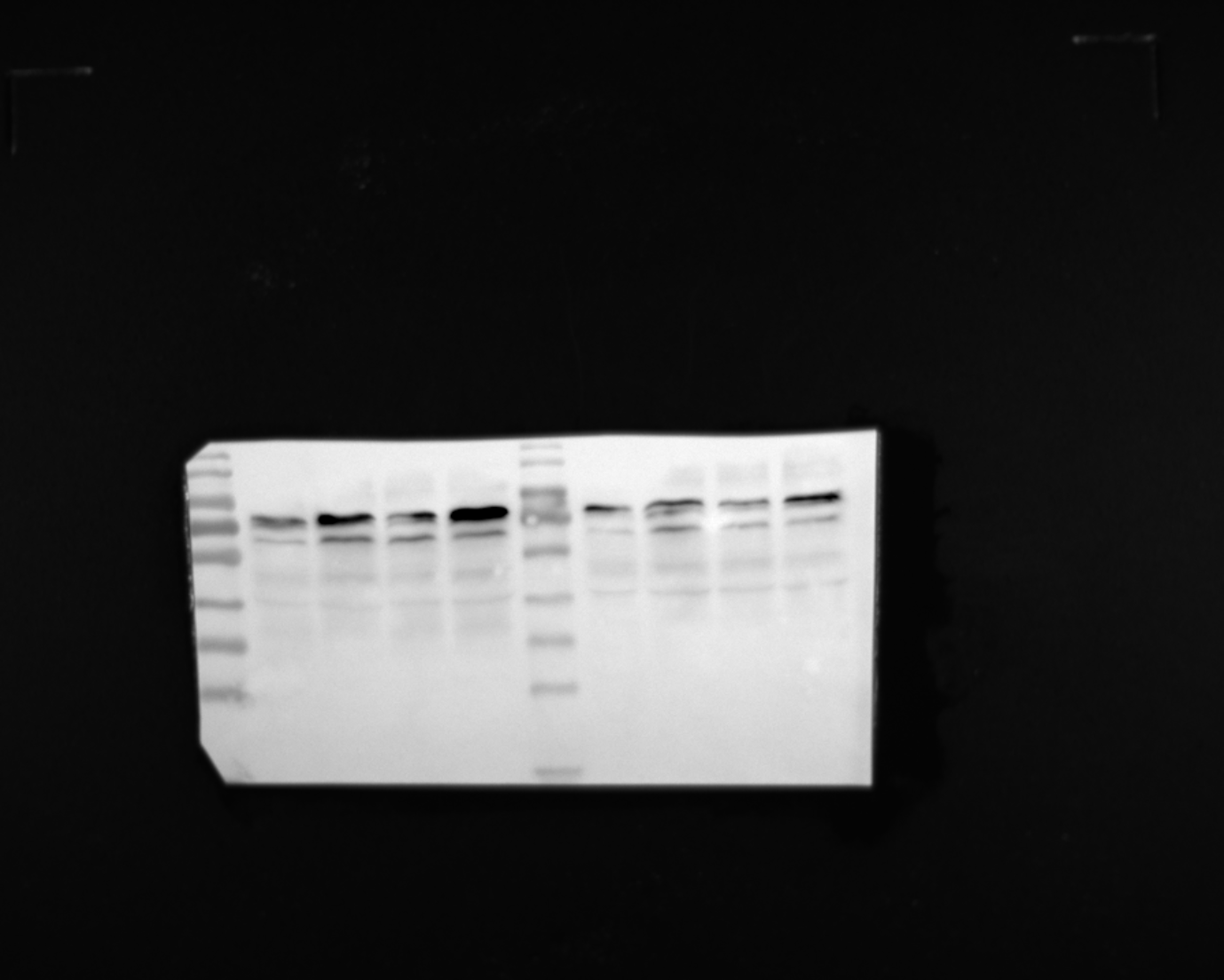

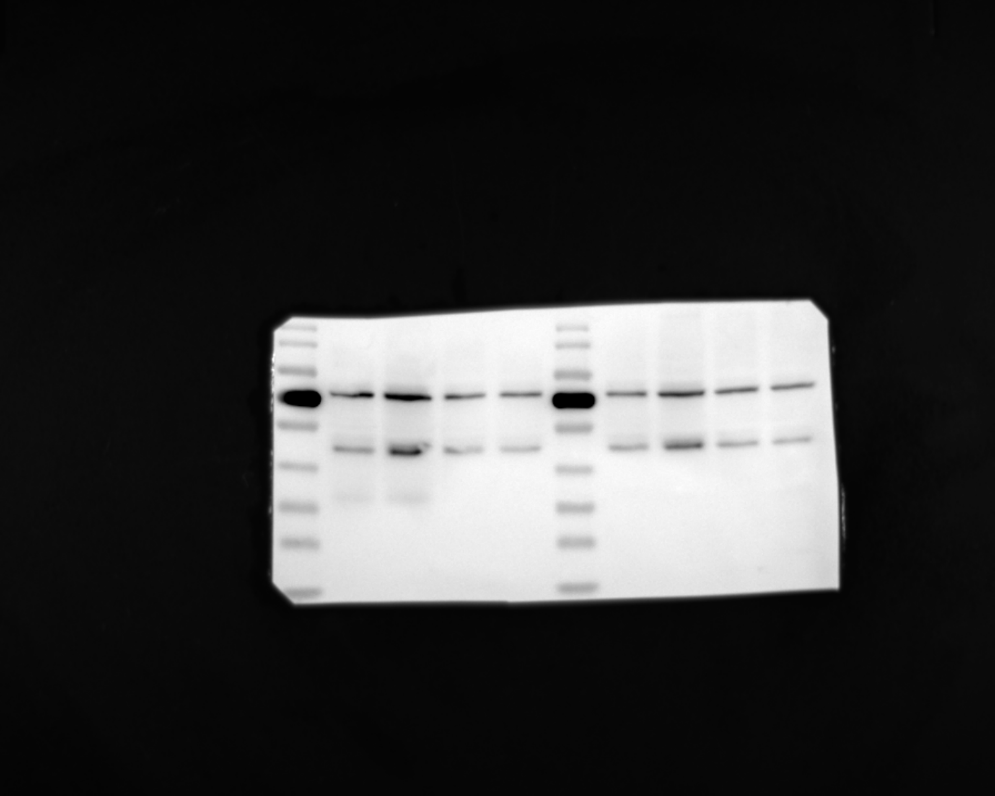


3

1

2

4

YY1

2

1

4

3

METTL3


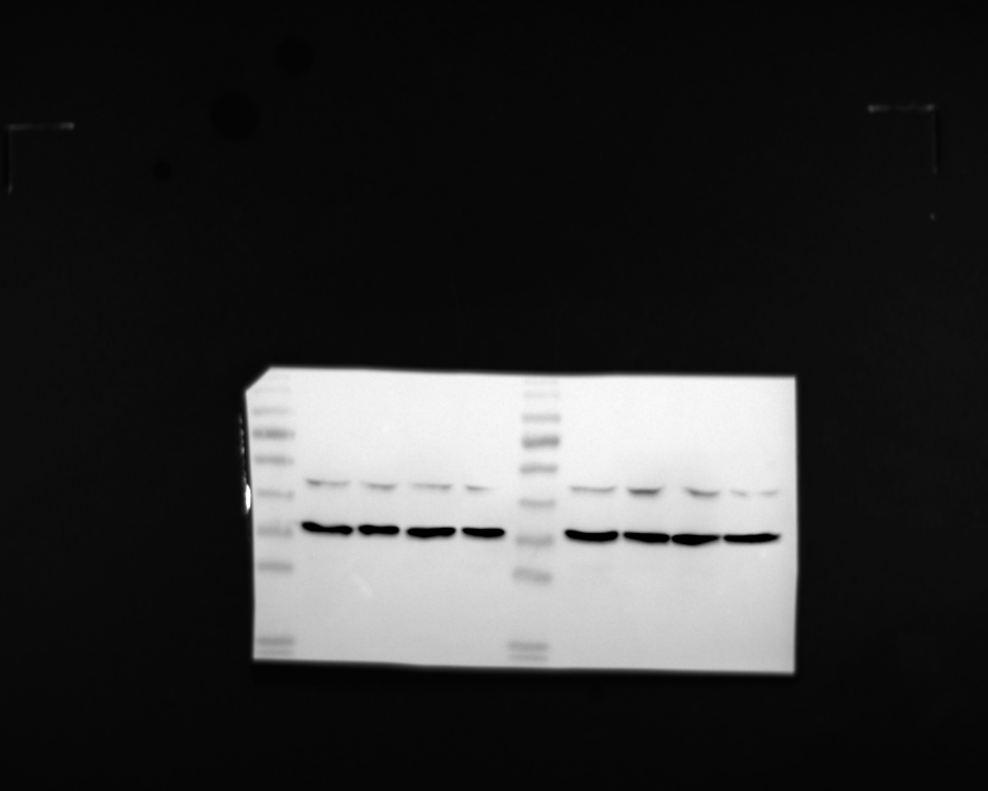


4

3

2

1

GAPDH

**Full and uncropped western blot for Figure 2 B**

Lanes 1, 2, 3, 4 are on the figure


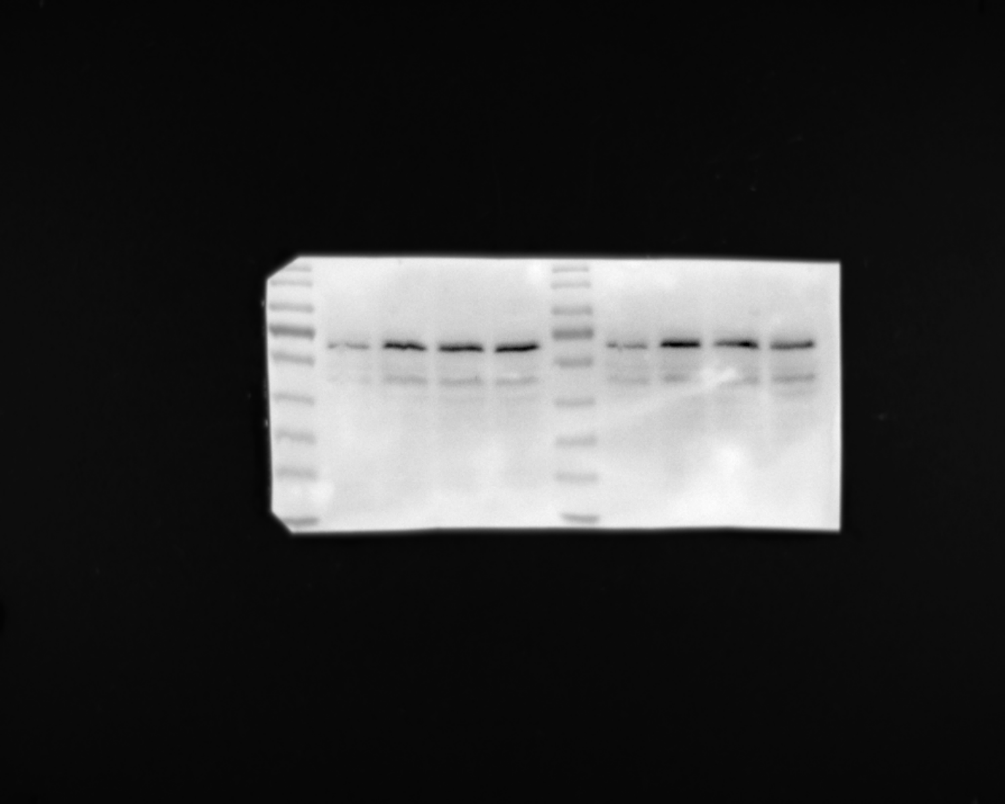

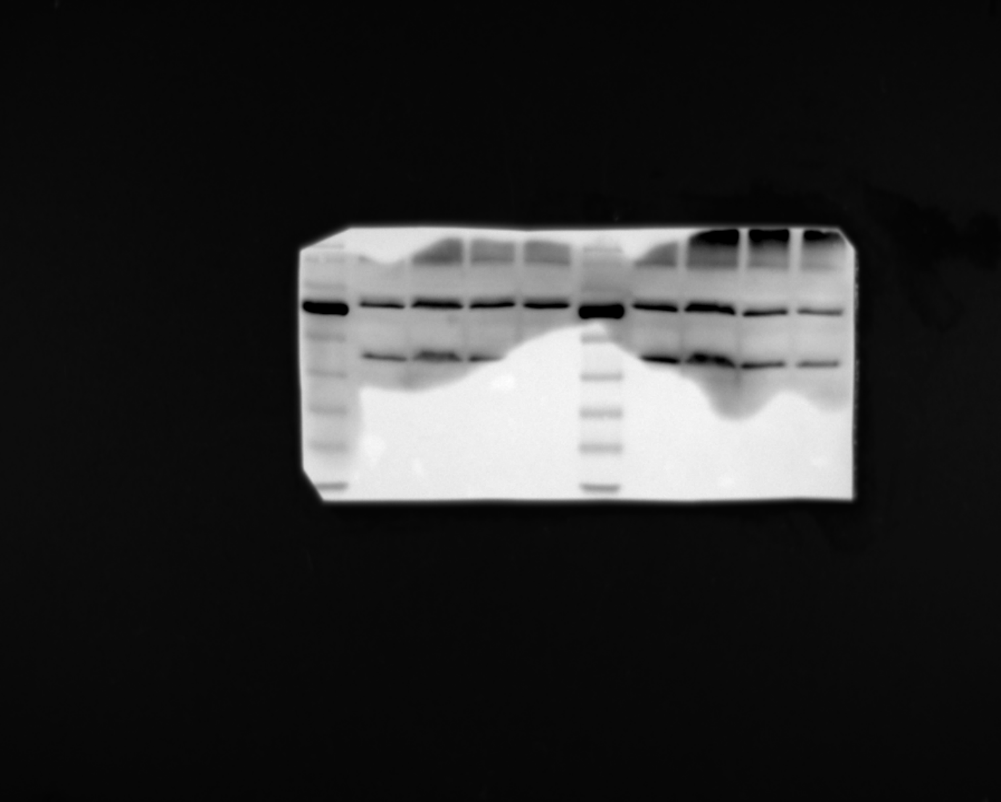


2

3

4

1

METTL3

2

3

4

1

YY1


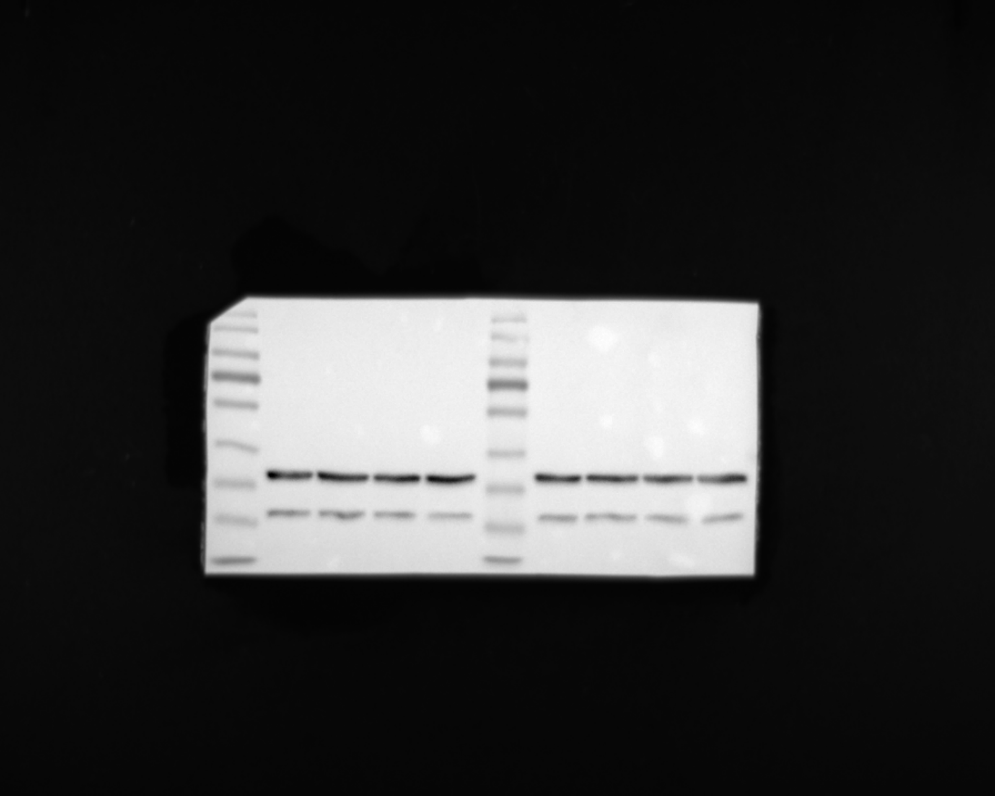


2

3

4

1

GAPDH

**Full and uncropped western blot for Figure 3A**

All the lanes are on the figure

IP

lysates


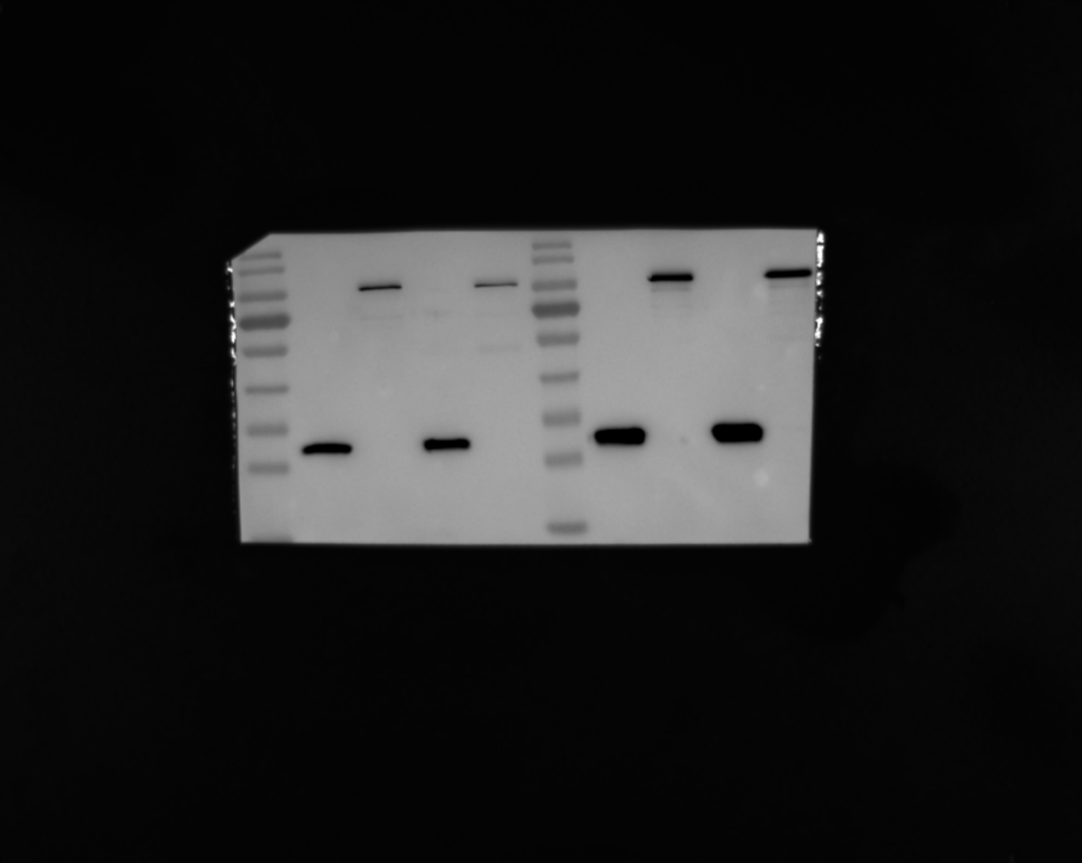


IP

lysates

Anti-EGFP


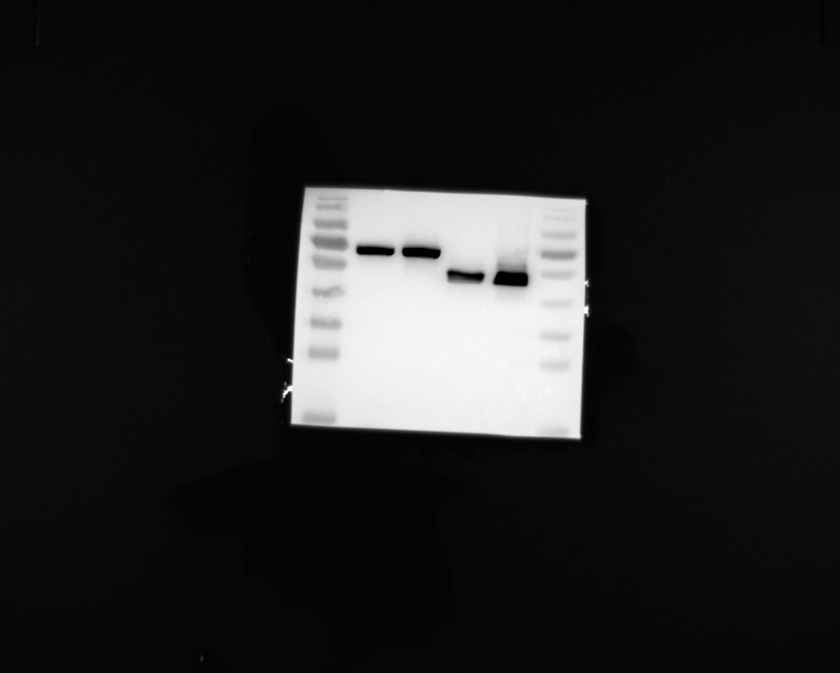

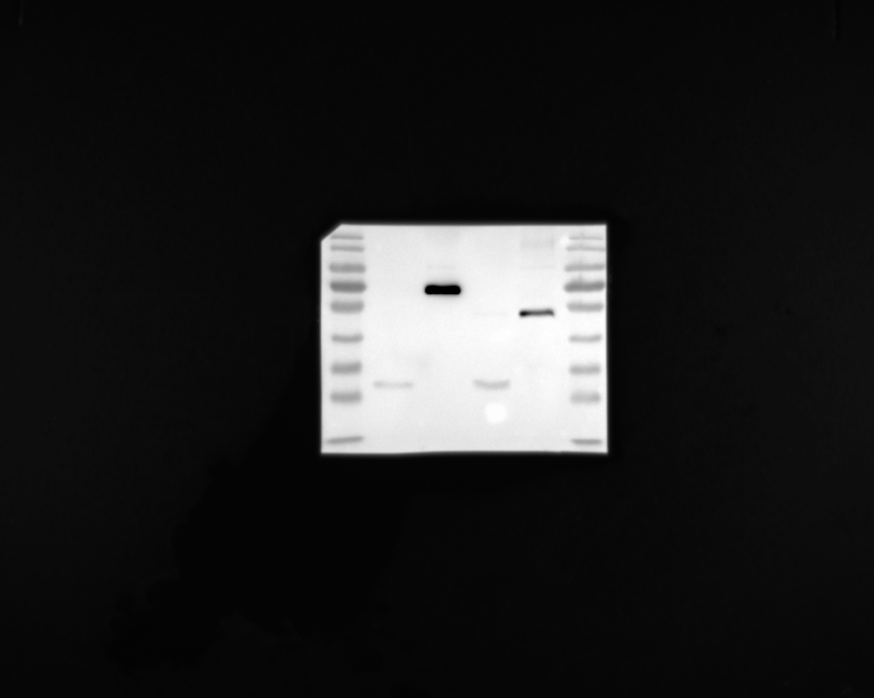


Anti-HA

**Full and uncropped western blot for Figure 3B**

All the lanes are on the figure

IP

lysates


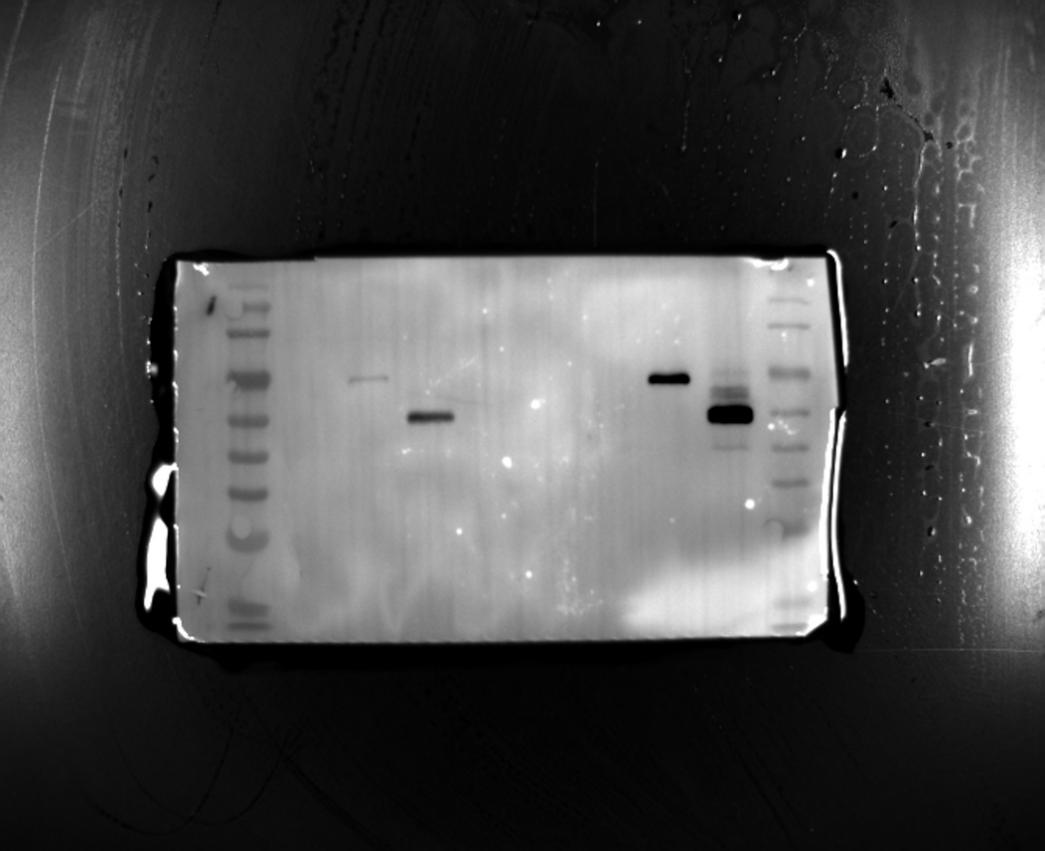


Anti-HA

IP

lysates


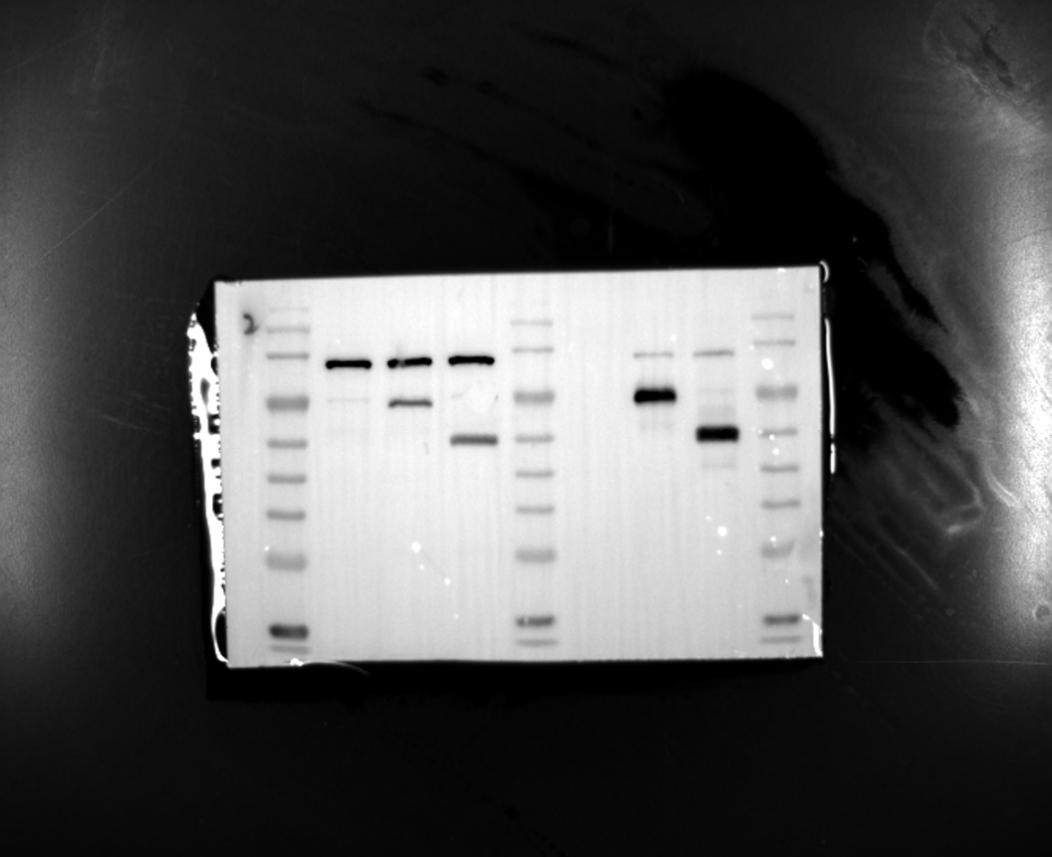


Anti-EGFP

**Full and uncropped western blot for Figure 3 E**

The lanes are on the figure

lysates

IP

**
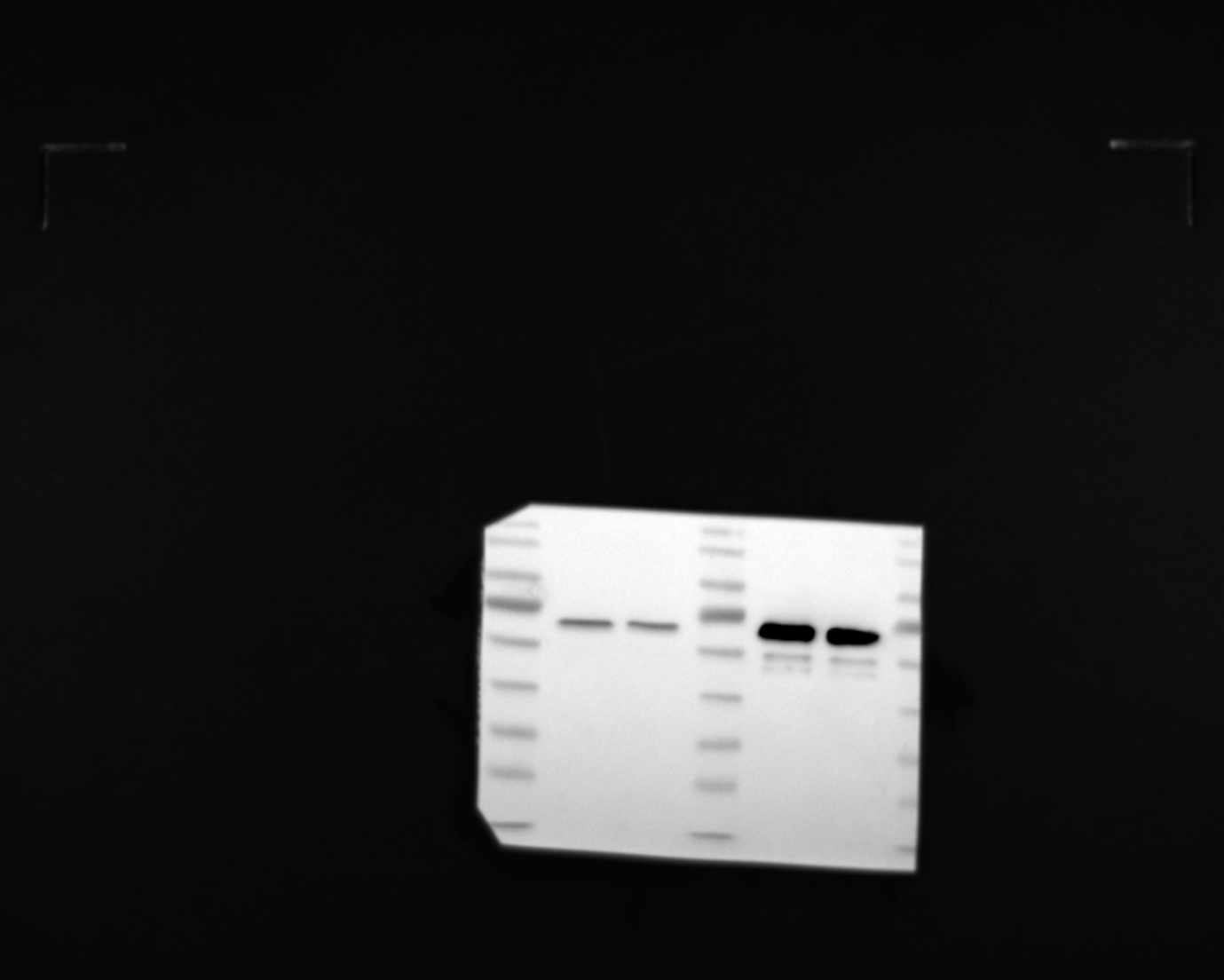
**

IP

Anti-YY1

**
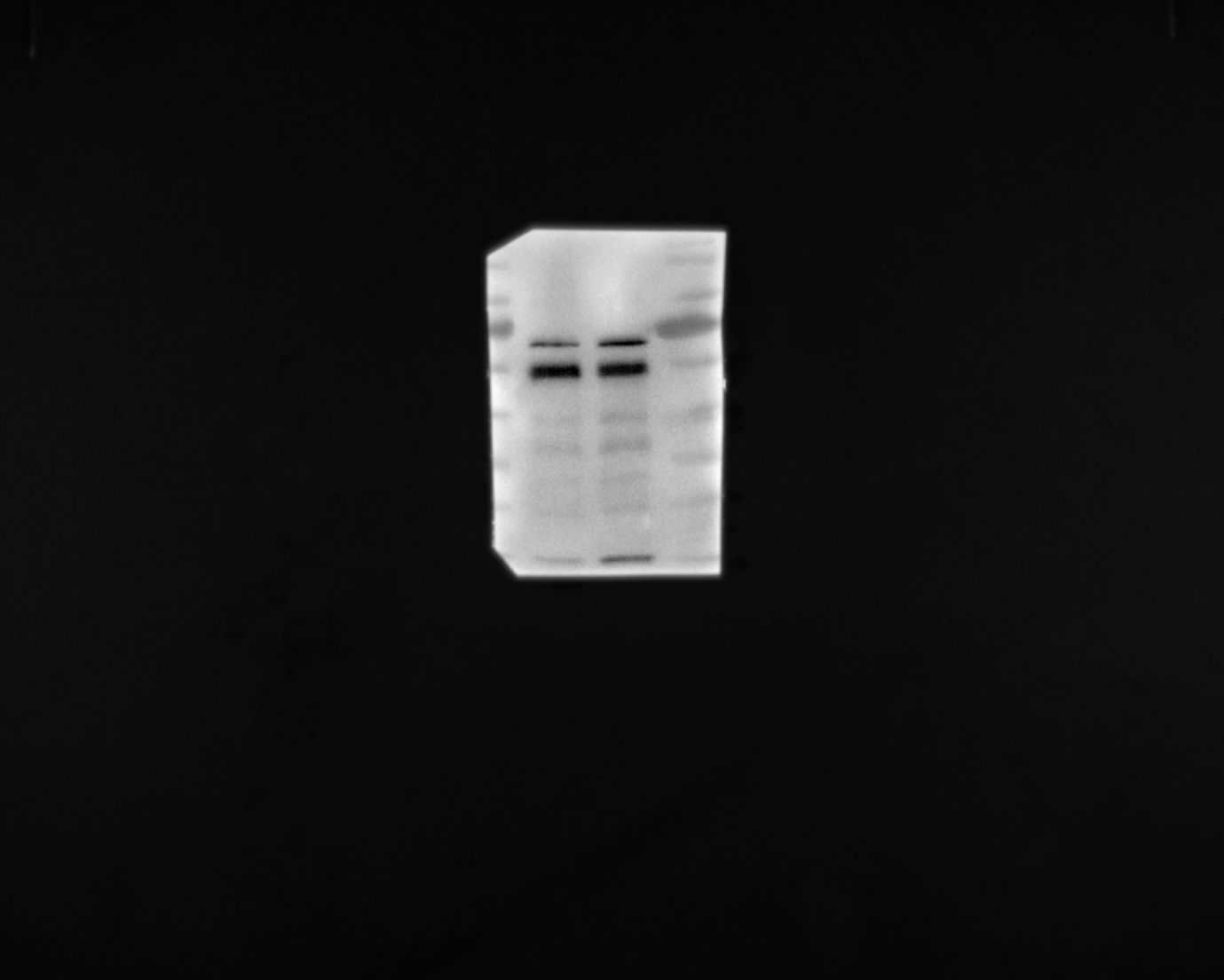
**

Anti-pan-acetylation

**Full and uncropped western blot for Figure 3 F**

Lanes 1, 2, 3, 4 are on the figure


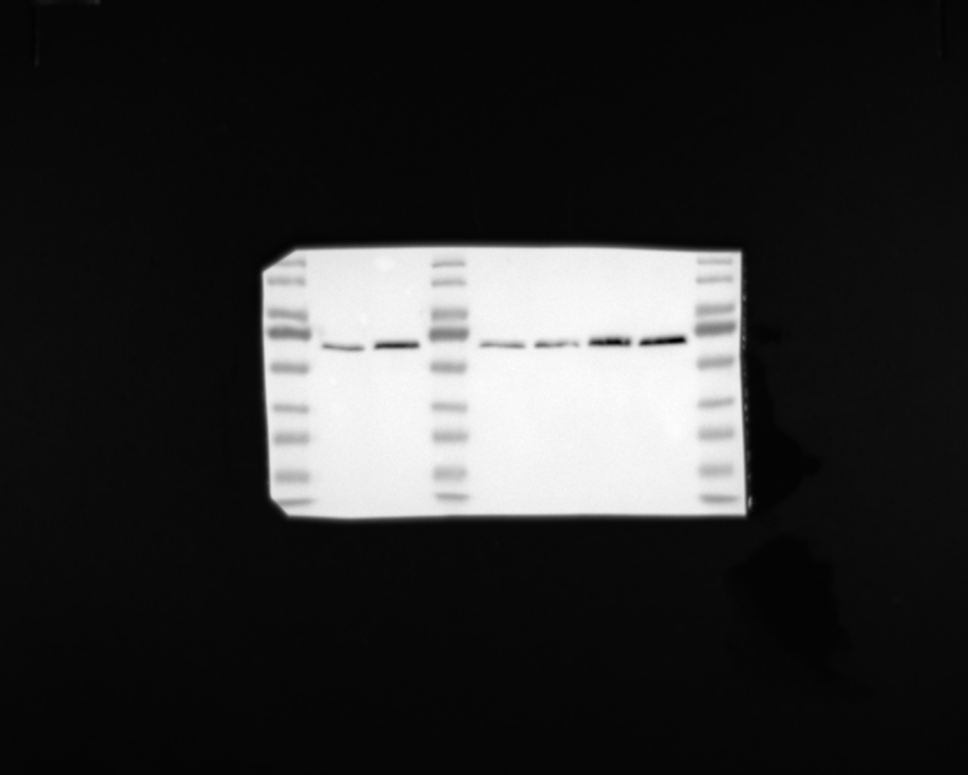

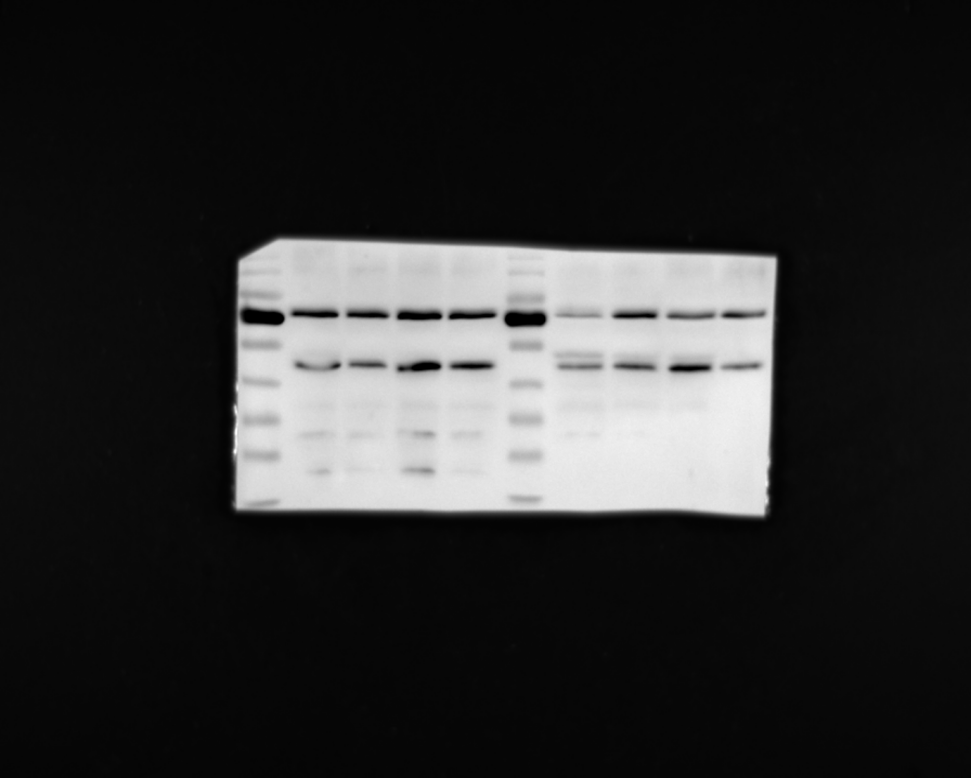


4

3

2

1

METTL3

3

4

1

2

YY1


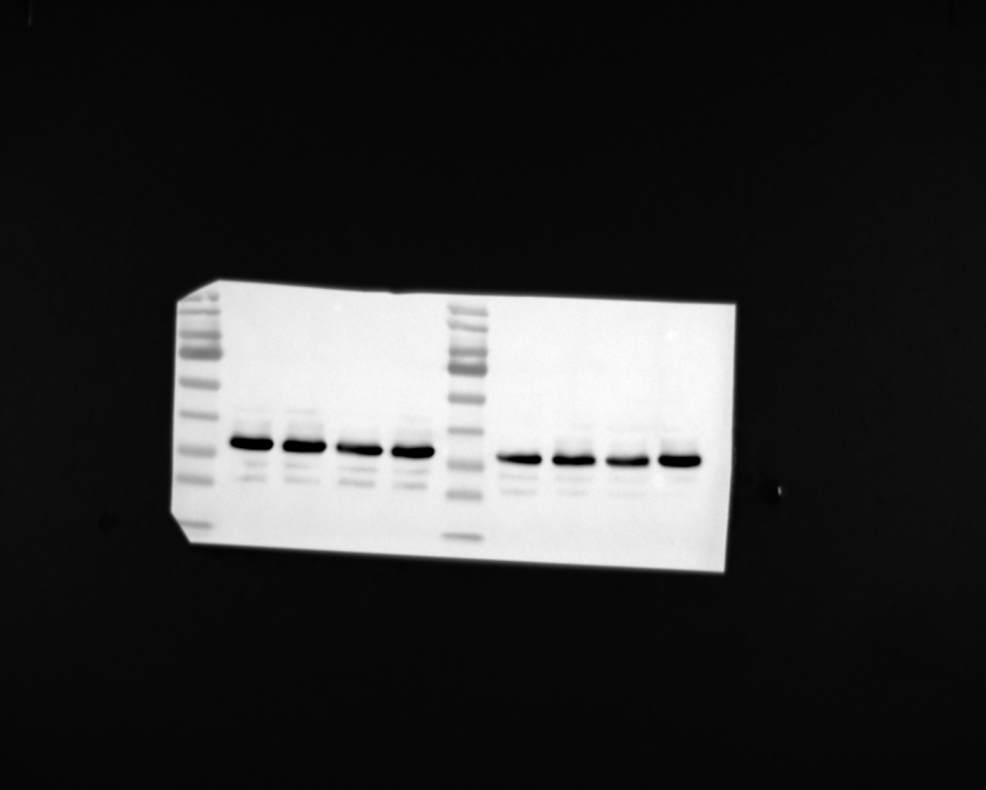


4

3

2

1

GAPDH

**Full and uncropped western blot for Figure 3 G**

Lanes 1, 2, 3, 4 are on the figure


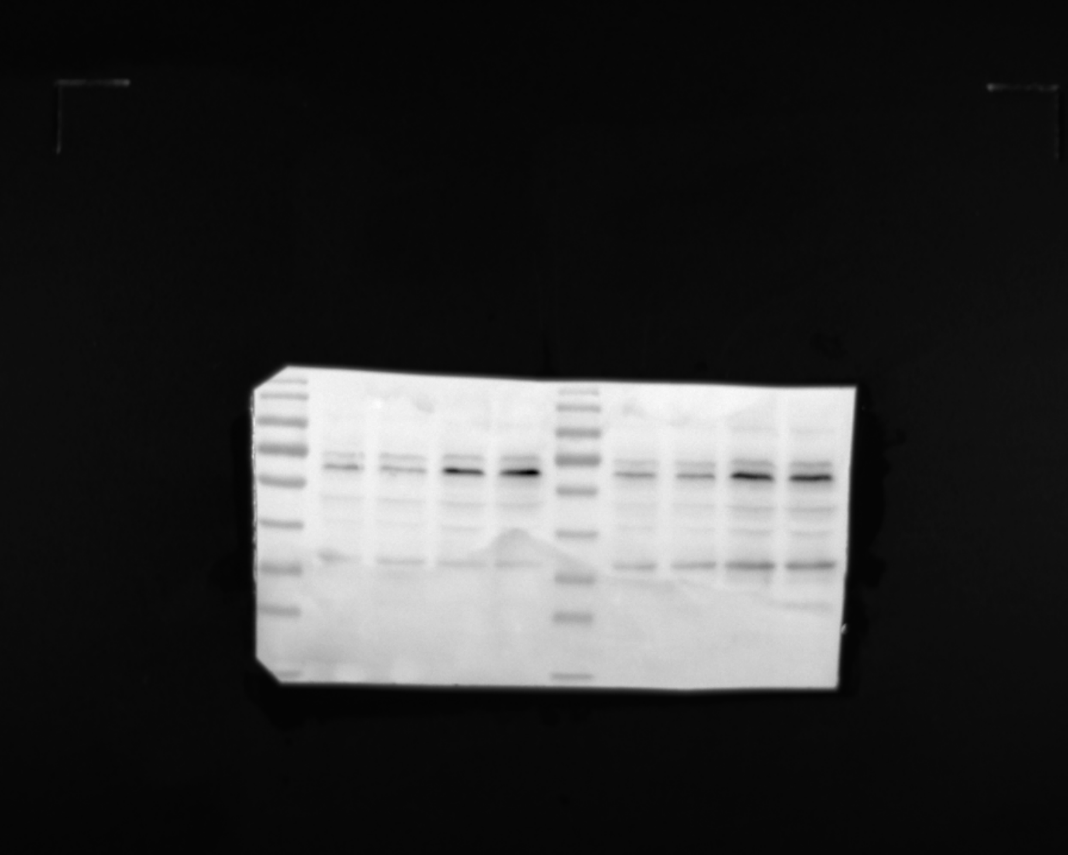

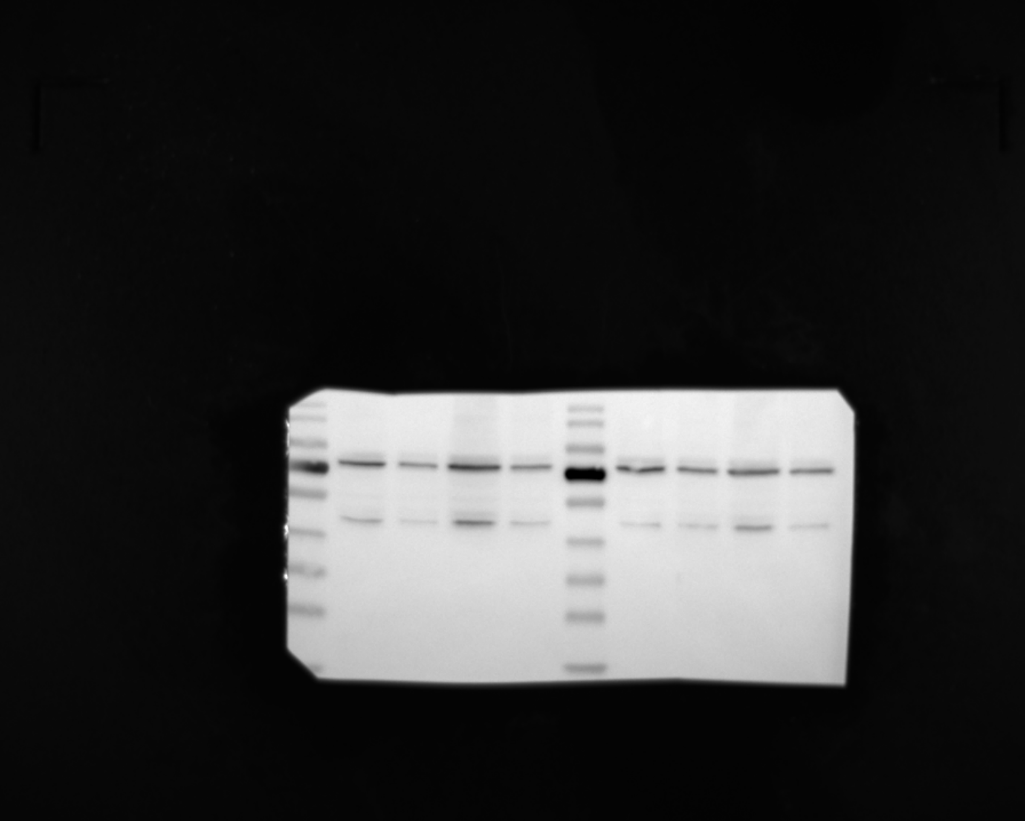


3

4

1

2

METTL3

2

3

4

1

YY1


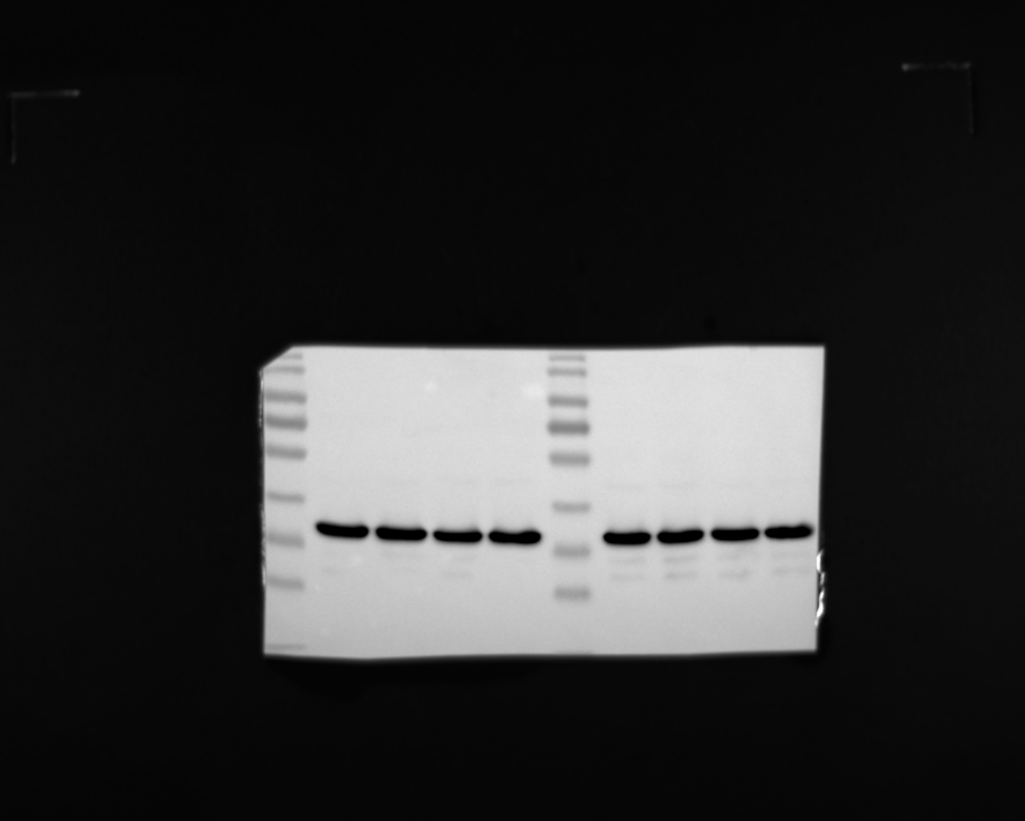


2

4

3

1

GAPDH

**Full and uncropped western blot for Figure 6 B (HDAC1)**

Lanes 1, 2, 3, are on the figure

IP

lysates


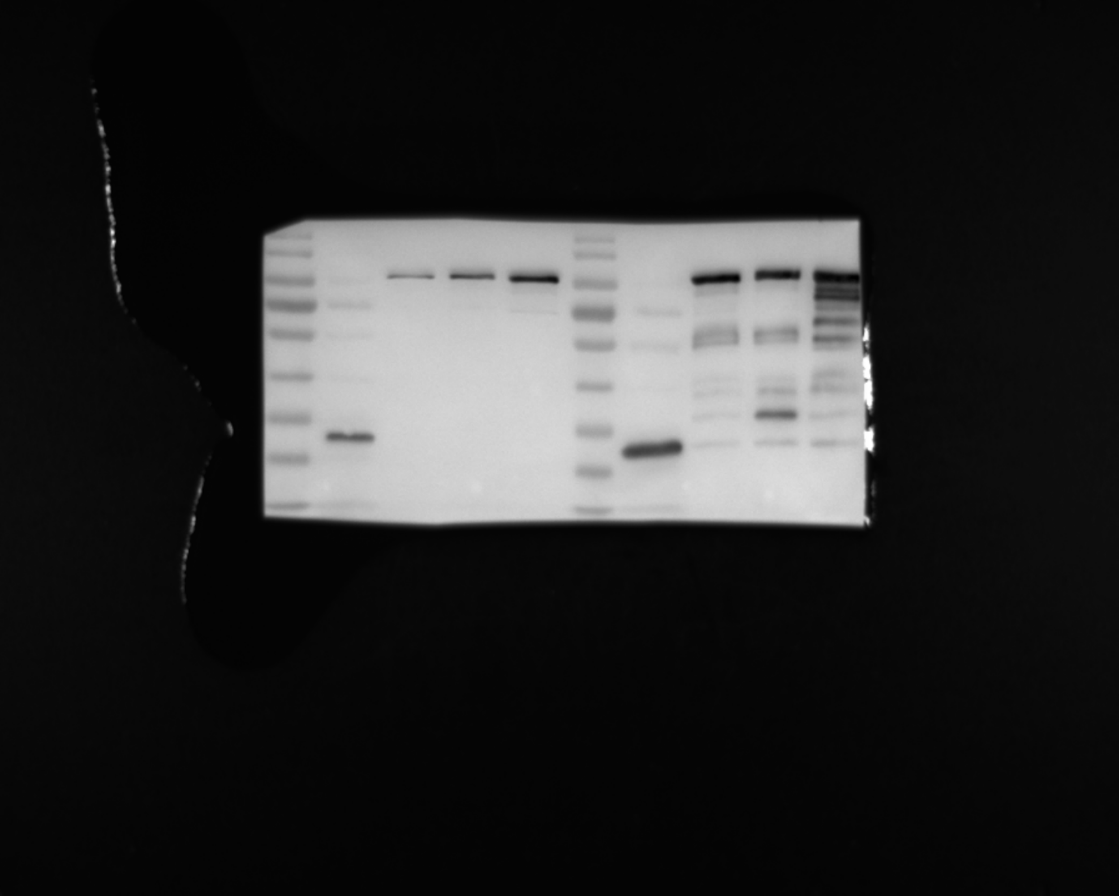

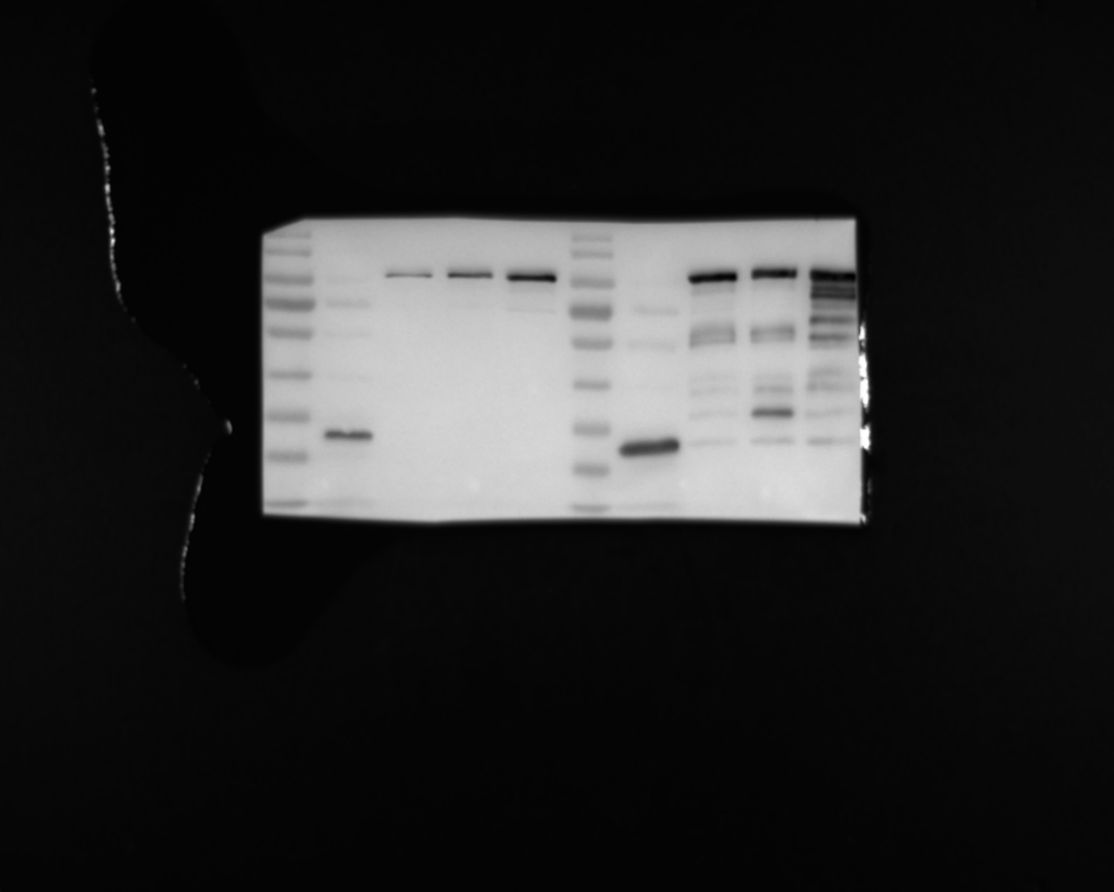


IP

lysates

2

1

3

1

2

3

Anti-EGFP


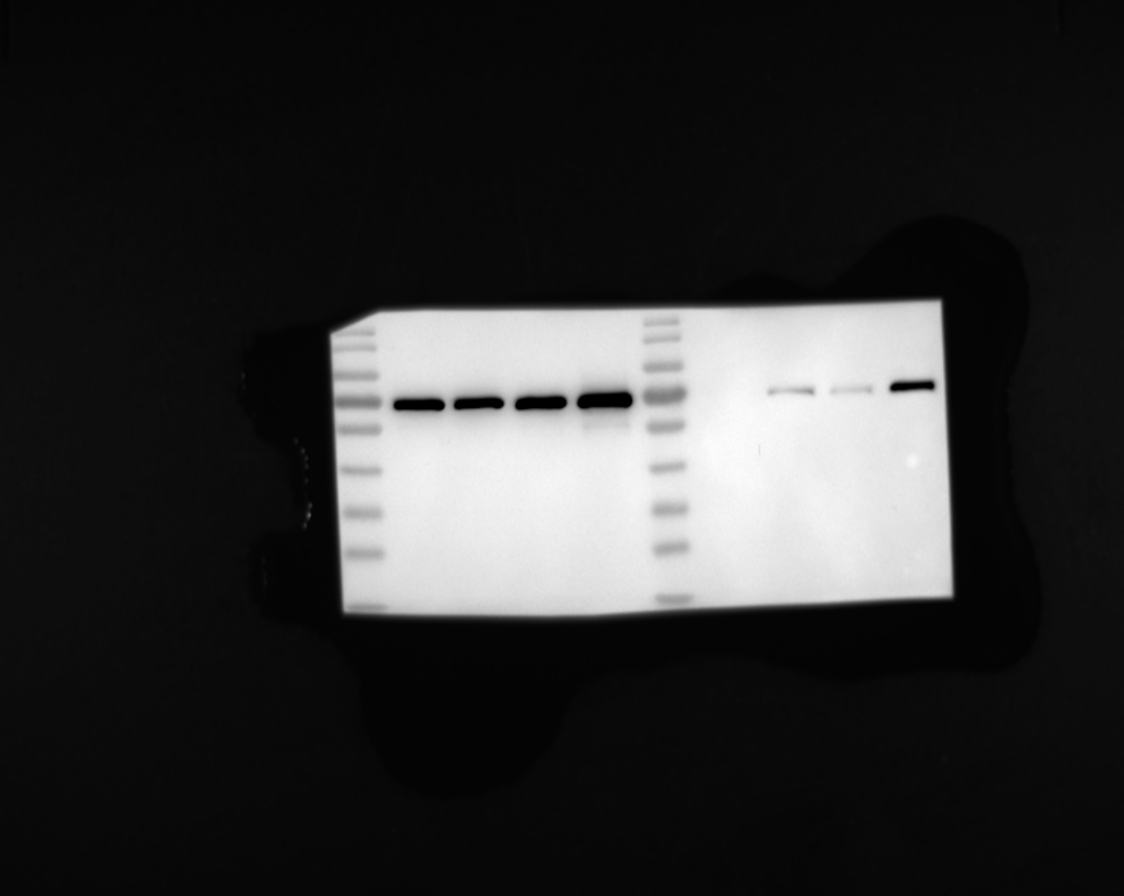

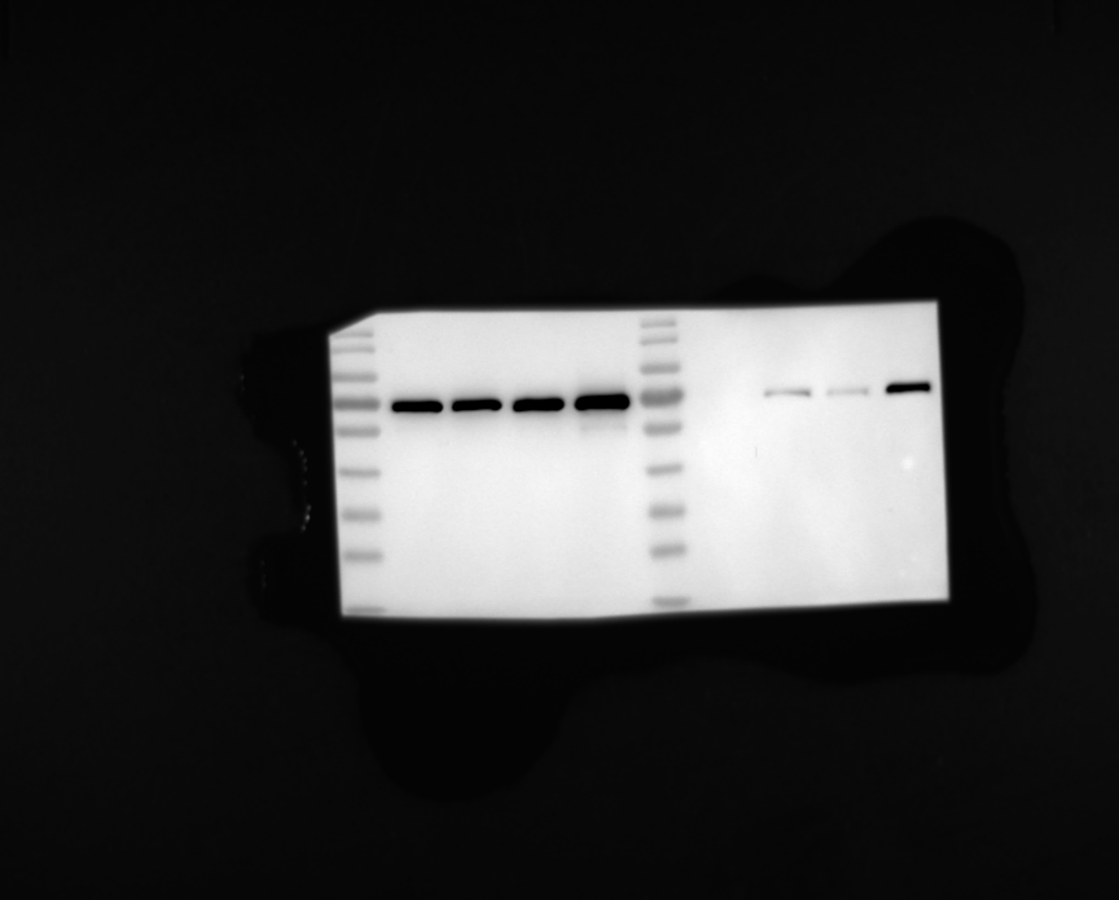


1

2

3

2

3

1

Anti-HA

**Full and uncropped western blot for Figure 6 B (HDAC3)**

Lanes 1, 2, 3, are on the figure

IP

lysates


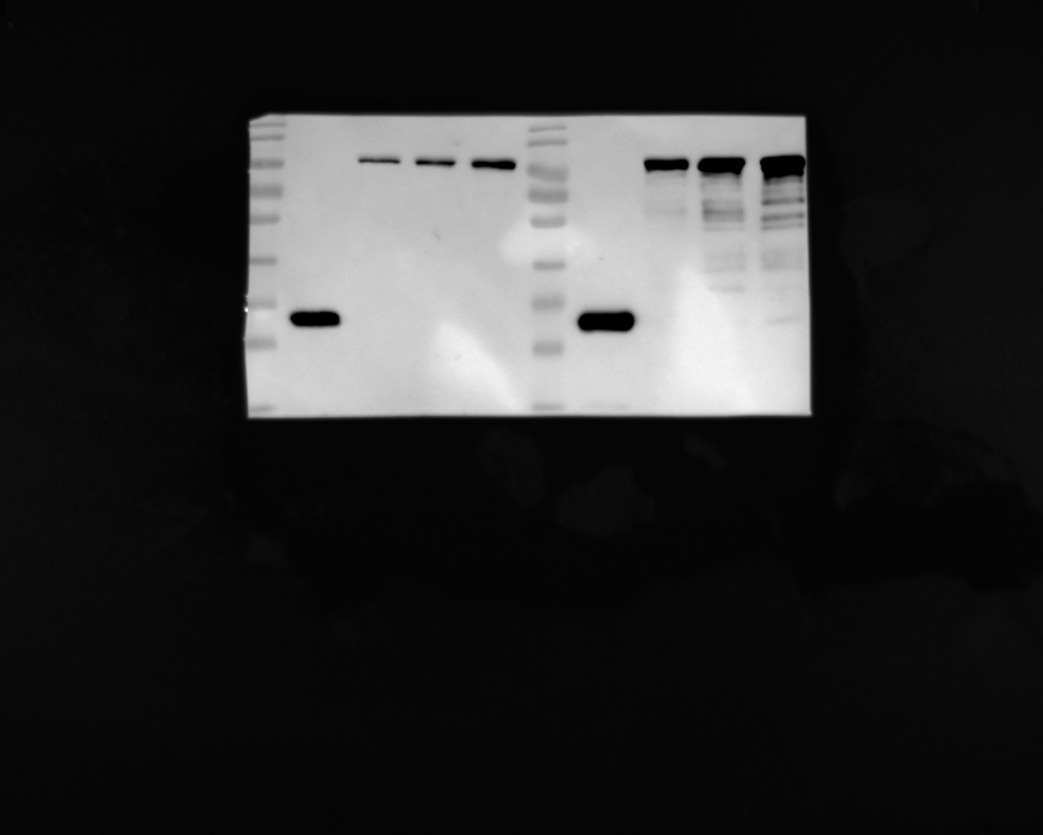

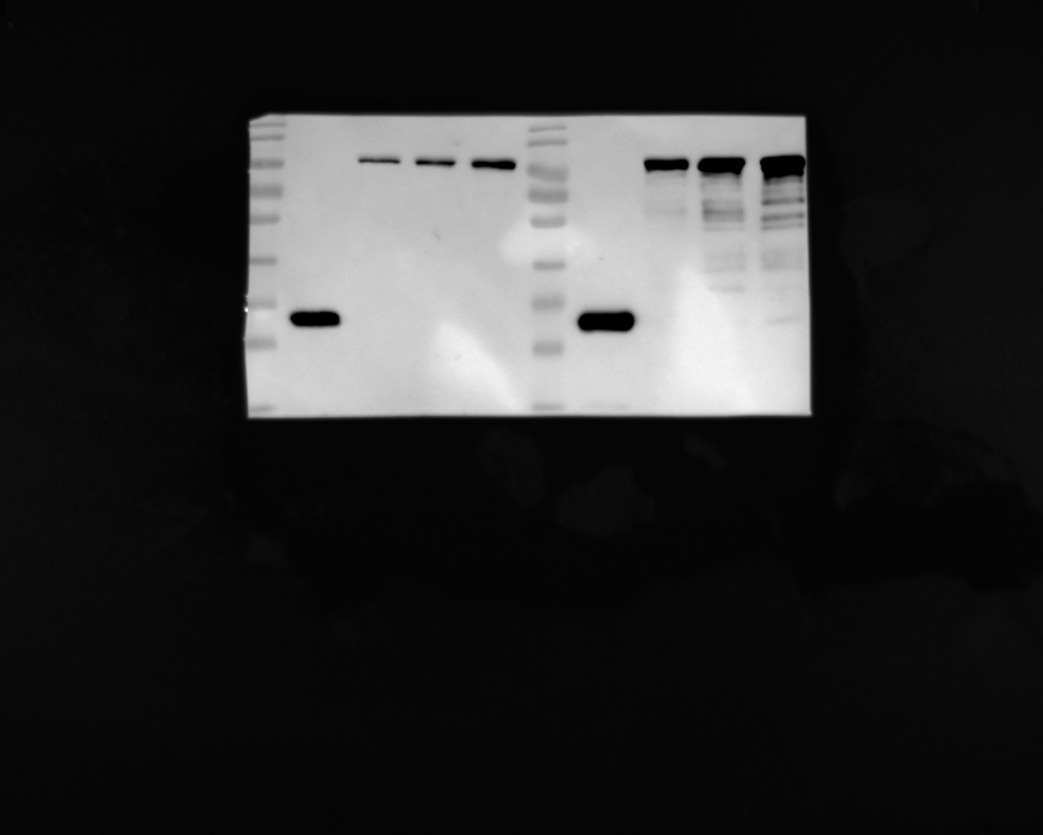


1

2

3

1

2

3

Anti-EGFP

IP

lysates


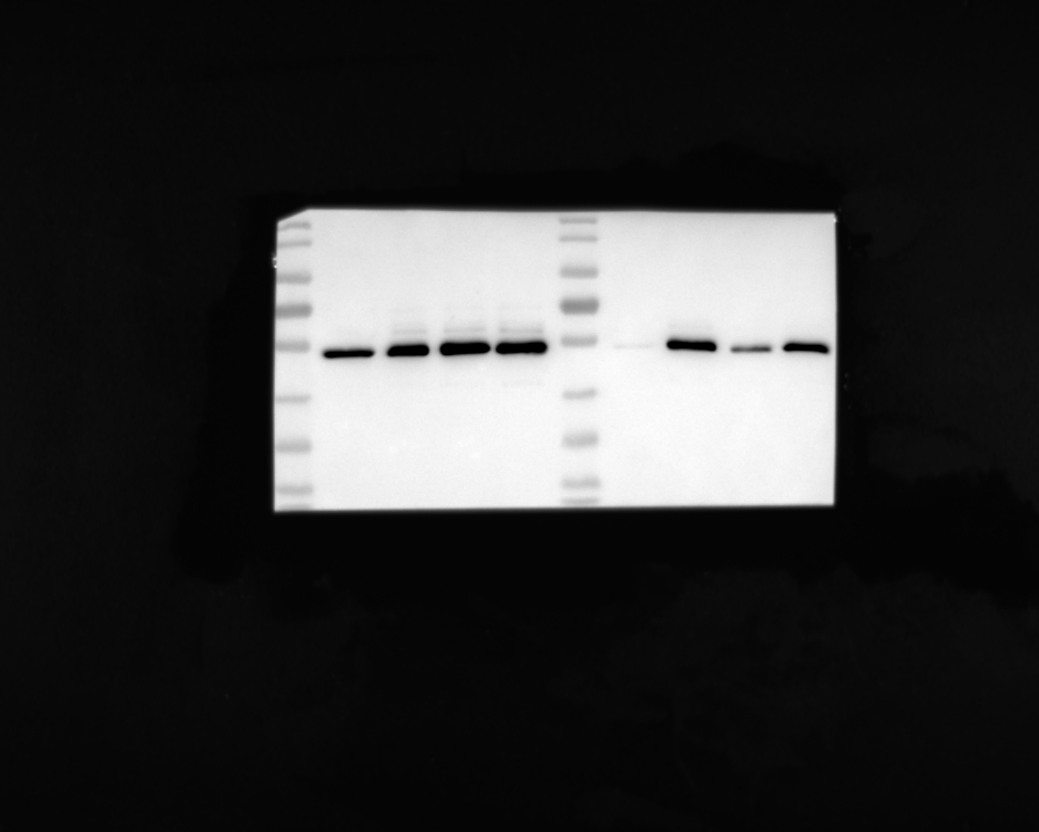

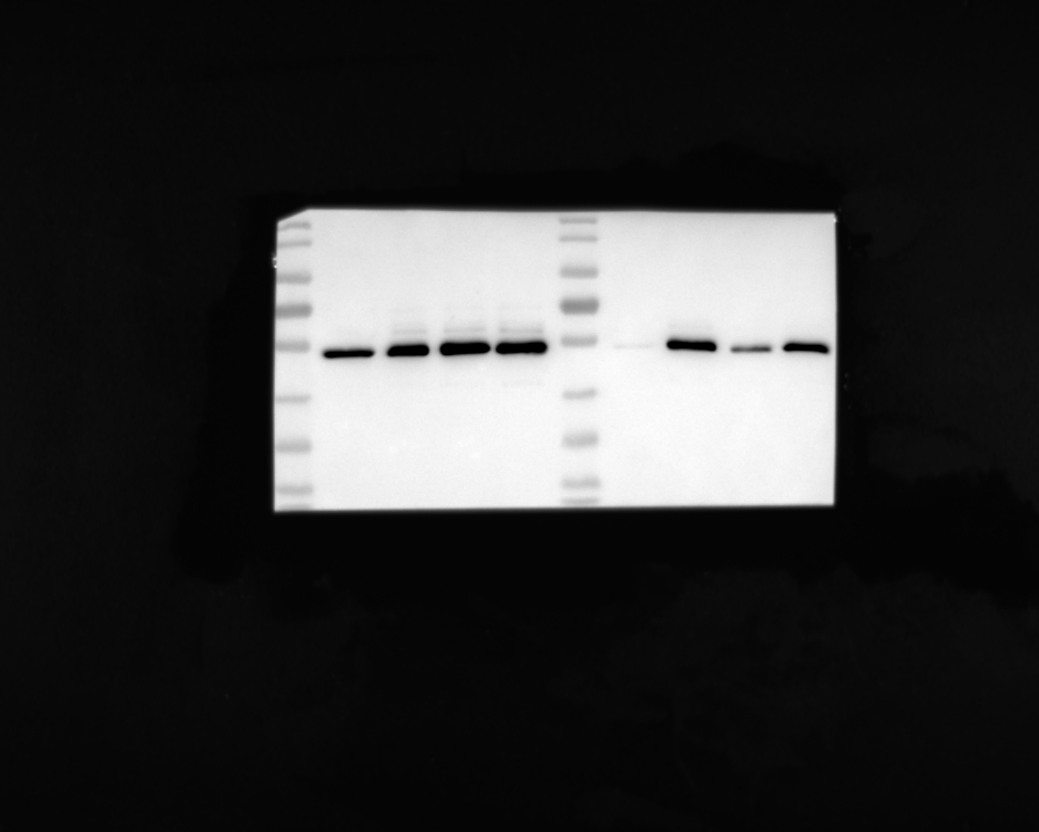


3

3

1

2

2

1

Anti-HA

**Full and uncropped western blot for Figure S3 A**

Lanes 1, 2, 3, are on the figure


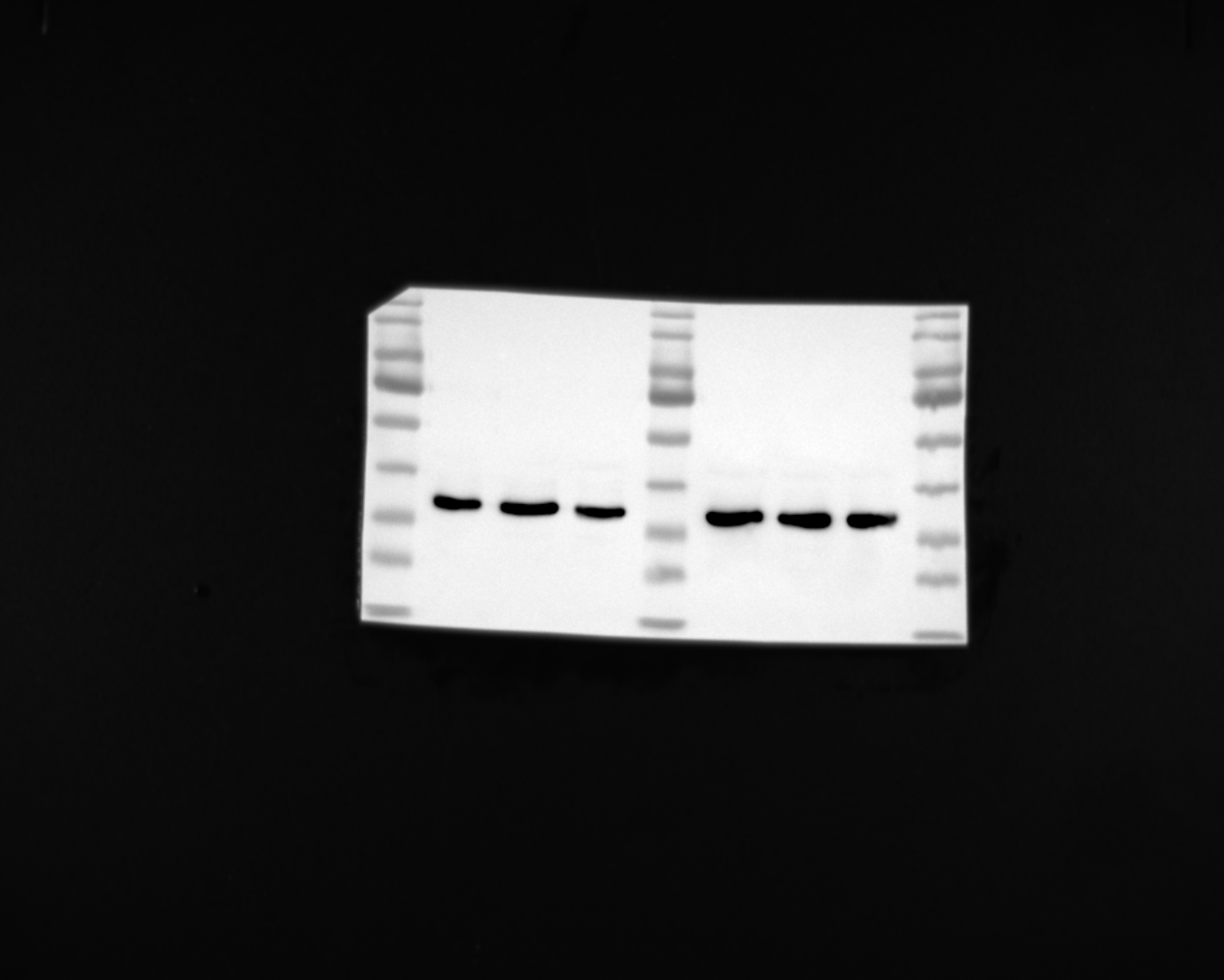

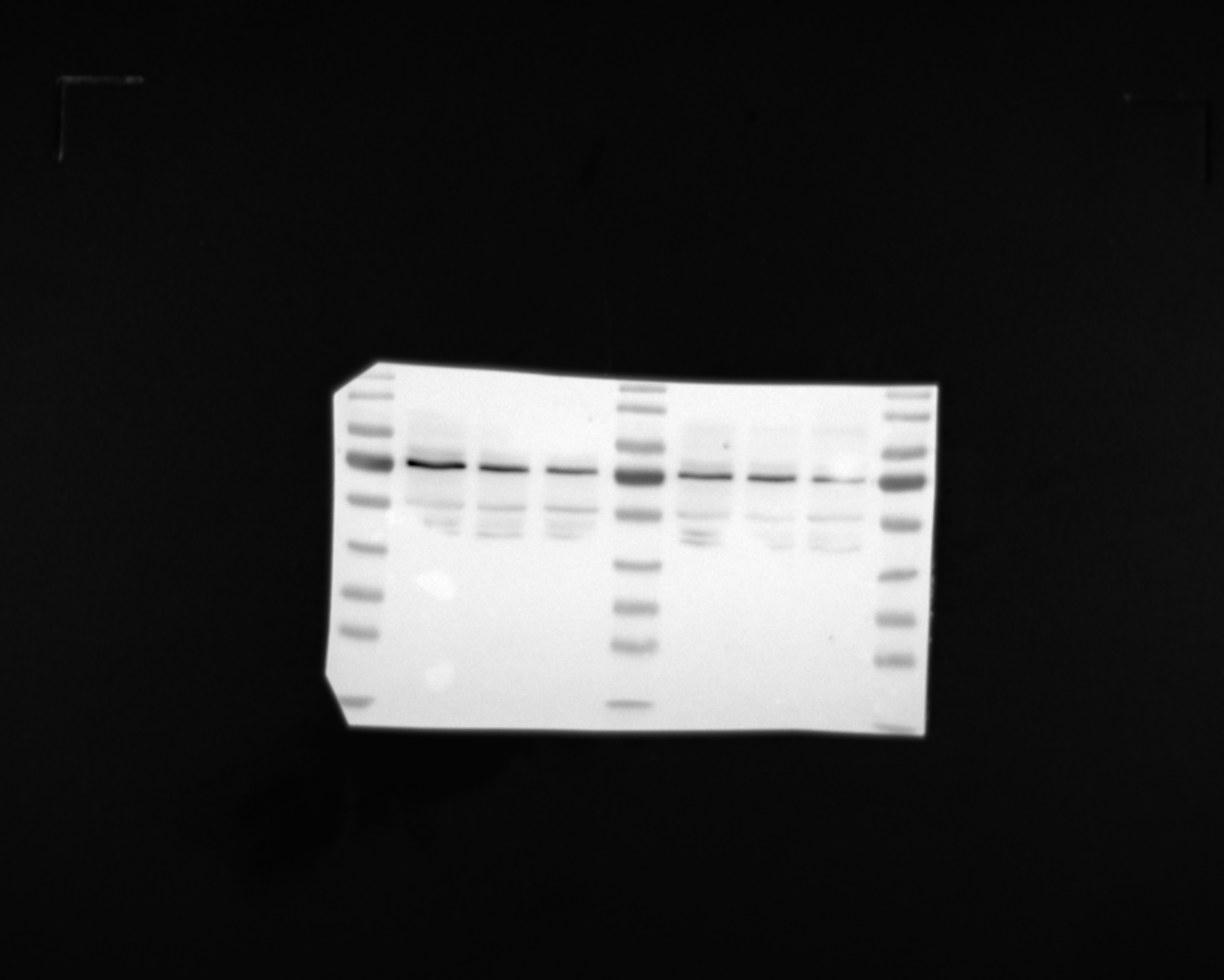


1

METTL3

3

2

GAPDH

1

2

3

**Full and uncropped western blot for Figure S3 B**

Lanes 1, 2, 3, are on the figure


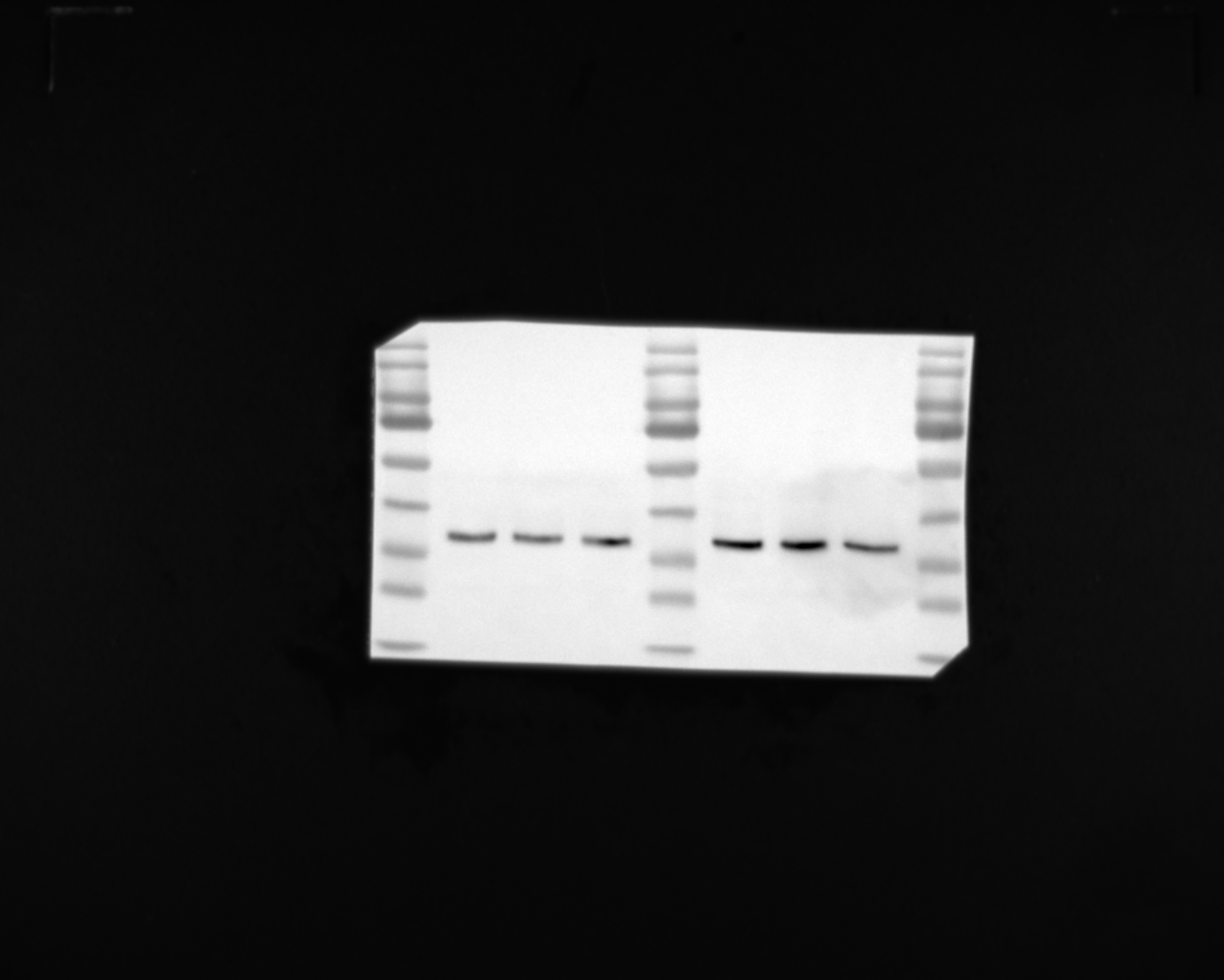

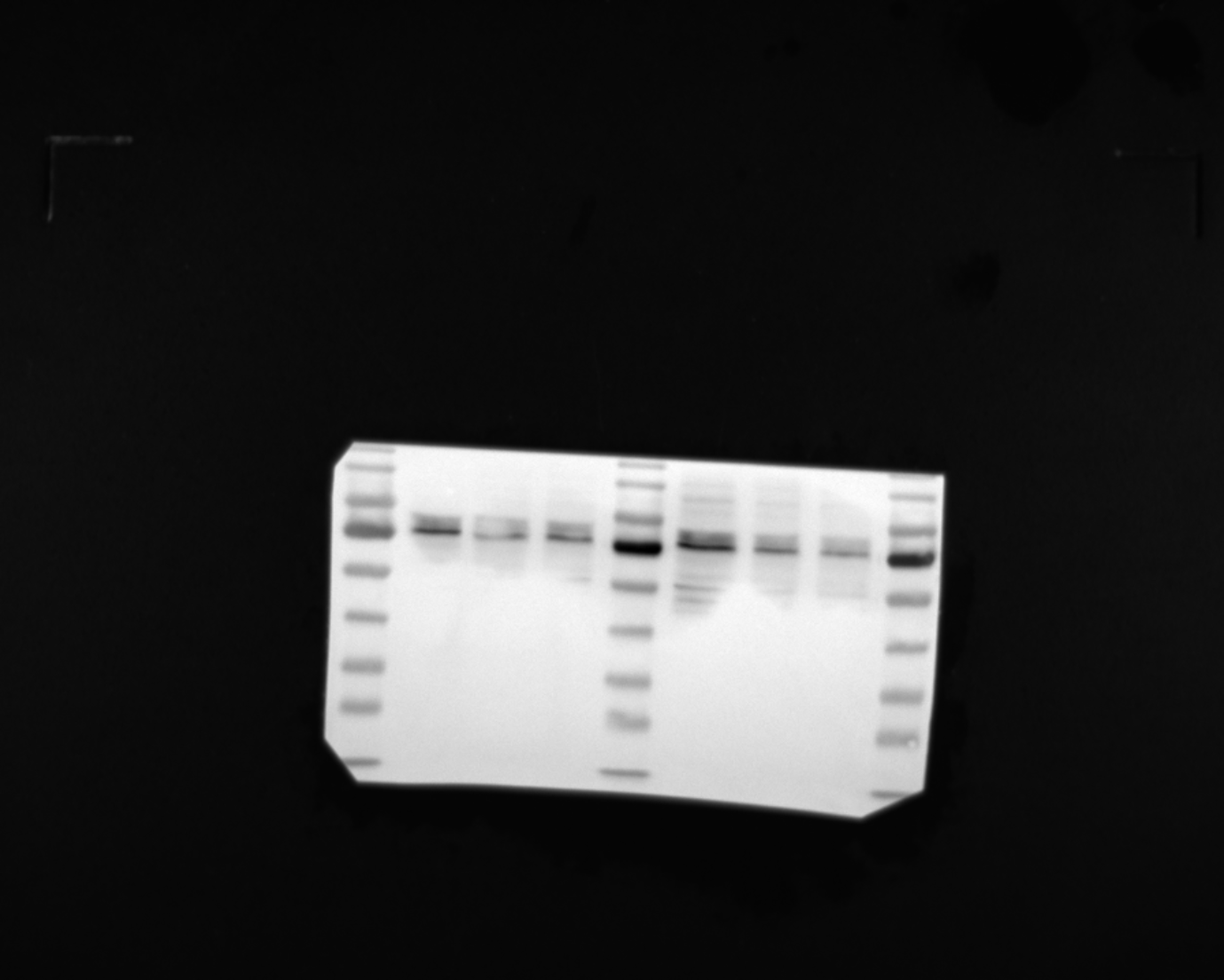


GAPDH

METTL3

3

2

1

3

1

2
